# Aberrant amino acid metabolism promotes neurovascular reactivity in rosacea

**DOI:** 10.1172/jci.insight.161870

**Published:** 2022-11-22

**Authors:** Tangxiele Liu, Wenqin Xiao, Mengting Chen, Rui Mao, Xu San, Qinqin Peng, Zhixiang Zhao, Qian Wang, Hongfu Xie, Zhili Deng, Ji Li

**Affiliations:** 1 Department of Dermatology,; 2 Hunan Key Laboratory of Aging Biology, and; 3 National Clinical Research Center for Geriatric Disorders, Xiangya Hospital, Central South University, Changsha, China.; 4 Hunan Binsis Biotechnology Co., Ltd, Changsha, China.

**Keywords:** Dermatology, Metabolism, Amino acid metabolism, Nitric oxide, Skin

## Abstract

Rosacea is a chronic skin disorder characterized by abnormal neurovascular and inflammatory conditions on the central face. Despite increasing evidence suggesting that rosacea is associated with metabolic disorders, the role of metabolism in rosacea pathogenesis remains unknown. Here, via a targeted metabolomics approach, we characterized significantly altered metabolic signatures in patients with rosacea, especially for amino acid-related metabolic pathways. Among these, glutamic acid and aspartic acid were highlighted and positively correlated with the disease severity in patients with rosacea. We further demonstrated that glutamic acid and aspartic acid can facilitate the development of erythema and telangiectasia, typical features of rosacea, in the skin of mice. Mechanistically, glutamic acid and aspartic acid stimulated the production of vasodilation-related neuropeptides from peripheral neurons and keratinocytes and induced the release of nitric oxide from endothelial cells and keratinocytes. Interestingly, we provided evidence showing that doxycycline can improve the symptoms of patients with rosacea possibly by targeting the amino acid metabolic pathway. These findings reveal that abnormal amino acid metabolism promotes neurovascular reactivity in rosacea and raise the possibility of targeting dysregulated metabolism as a promising strategy for clinical treatment.

## Graphical abstract

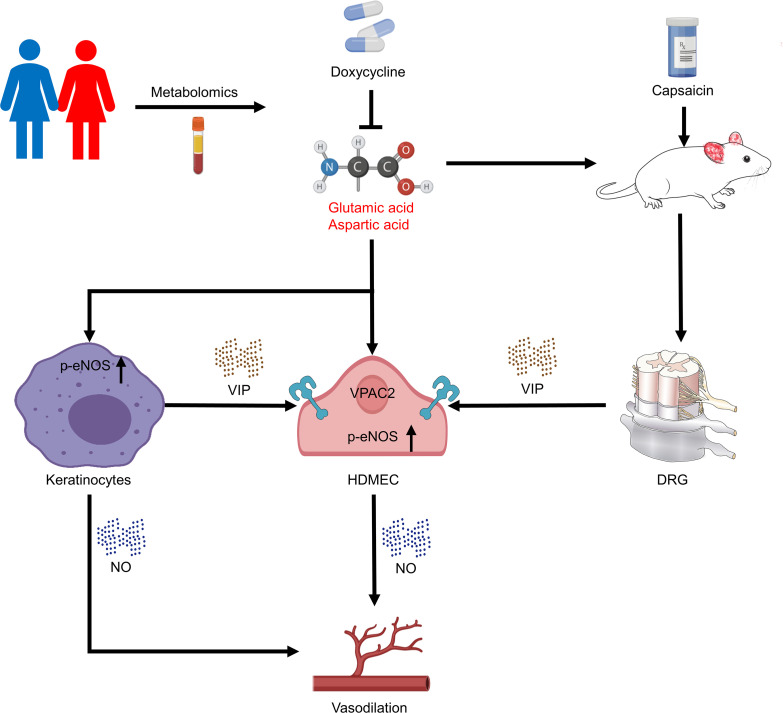

## Introduction

Rosacea is a commonly chronic, progressive skin disease that presents with aberrant neurovascular and inflammatory conditions on the central face, affecting about as much as 5.5% of the global population (1, 2). Variable clinical manifestations, including transient or persistent erythema, telangiectasia, inflammatory papules/pustules, hyperplasia, and sensitive symptoms (such as stinging and burning), lead to psychological burden and decline in quality of life (3). Rosacea is often grossly underdiagnosed or misdiagnosed due to the multiple overlapping signs (4). There is still a lack of circulating indicators to evaluate the disease severity and condition. Current treatment strategies mainly focus on clinical assessment of phenotypic expressions. Sub-antimicrobial dose of doxycycline is the only FDA-approved oral medication for rosacea so far (5). Although rosacea can be controlled and managed, it does not have a cure.

Recent findings have indicated that rosacea is a combination of neurovascular dysfunction, innate and adaptive immune disorders, and metabolic abnormalities under certain genetic backgrounds. Triggers, including spicy food (capsaicin), heat, ultraviolet light, physical or chemical stimuli, and microbes, induce the release of various mediators from multiple cells, such as keratinocytes (for instance, cathelicidin, chemokines, and vascular endothelial growth factor), endothelial cells (nitric oxide and adhesion molecules), macrophages (inflammatory factors, matrix metalloproteinases, and nitric oxide), mast cells (matrix metalloproteinases and histamines), and T helper cells (interferons, tumor necrosis factor, and interleukins). Triggers can also communicate directly to the cutaneous nervous system, thus via neuropeptides resulting in the symptoms of rosacea (6–8). However, knowledge about specific pathophysiological mechanisms is still very limited.

Accumulating studies have highlighted metabolic disorders as an important risk factor for rosacea, yet the exact cause-and-effect relationship remains to be determined. The frequency of obesity, insulin resistance, and dyslipidemia is significantly higher in rosacea (9). Coronary artery disease risk factors are more common in patients with rosacea than controls (10). Therefore, we hypothesize that there is a dysregulation of multiple biochemical pathways in rosacea. Till now, no studies to our knowledge have systematically mapped the metabolic characteristics of rosacea. A deeper understanding of abnormal metabolism in rosacea may provide new insight for diagnosis and therapy.

Here, absolute quantitative metabolomics (11) was employed to analyze the serum metabolite changes in 57 patients with rosacea before and after treatment with doxycycline of sub-antimicrobial dose and 63 age-, sex-, and BMI-matched healthy controls. Metabolomics revealed markedly altered metabolic signatures in patients with rosacea, especially for amino acid–related metabolic pathways. Glutamic acid and aspartic acid were greatly increased and positively correlated with the disease severity in patients with rosacea, which was validated in another cohort. Our subsequent in vivo and in vitro results demonstrated that glutamic acid and aspartic acid can promote the formation of erythema and telangiectasia in mouse skin by facilitating the generation of vasodilation-related neuropeptides in peripheral neuron and keratinocytes and inducing the release of nitric oxide in endothelial cells and keratinocytes. Furthermore, by combining network pharmacology, metabolomics before and after doxycycline treatment, and transcriptomic analysis of liver in rats with doxycycline or vehicle treatment, we revealed that doxycycline can obviously alleviate the clinical symptoms of rosacea patients, possibly by targeting amino acid metabolic pathway. These findings reveal a key role of amino acid metabolism in the pathogenesis of rosacea and suggest targeting dysregulated metabolism might be a novel therapeutic avenue for rosacea.

## Results

### Serum metabolic profiles in rosacea identified by targeted metabolomics.

**Figure 1:**
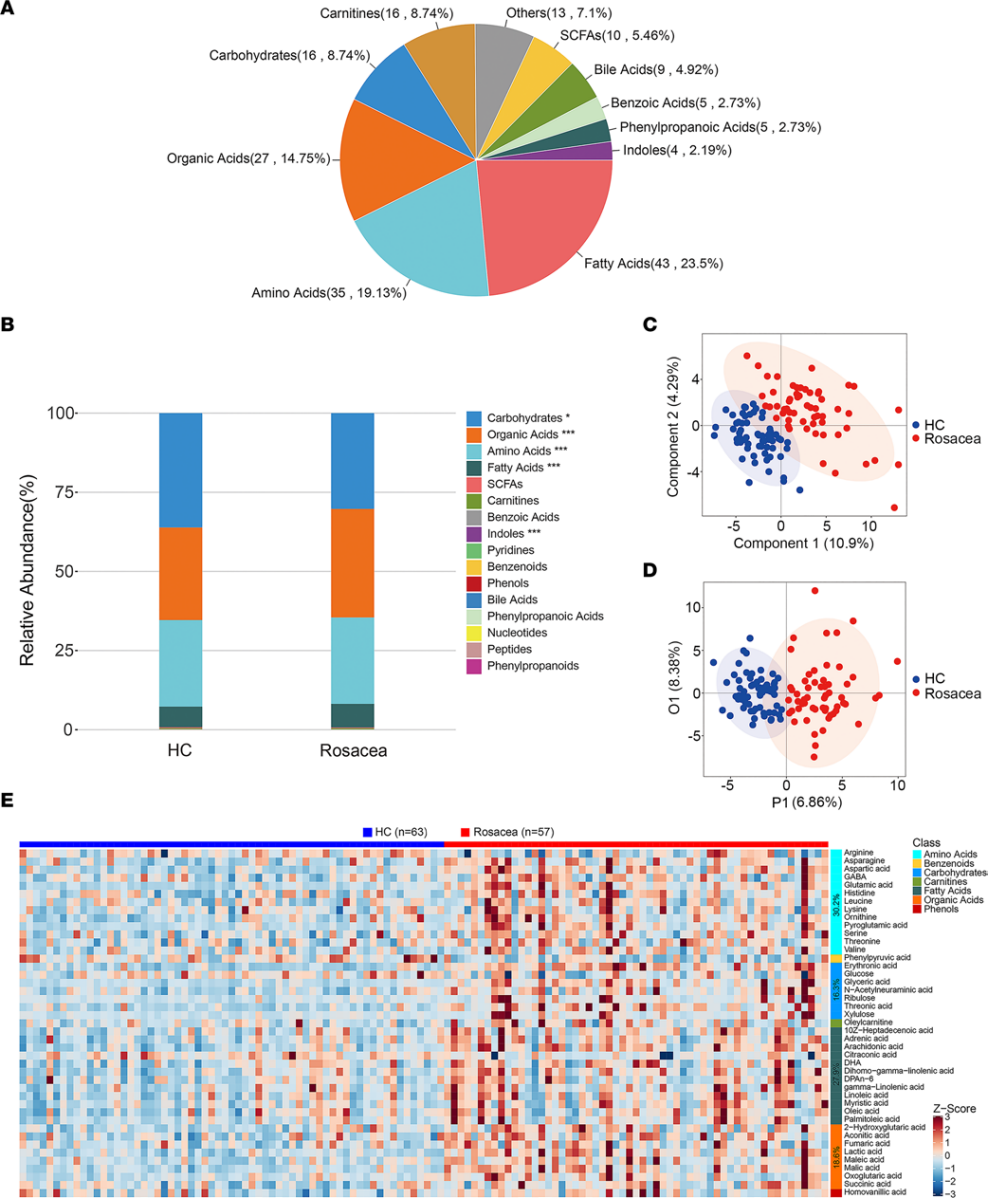
Serum metabolic profiling in patients with rosacea identified by targeted metabolomics. (**A**) The summary of detected metabolite counts in each metabolite class. (**B**) The relative abundance of each metabolite classes in different groups is shown in the stacked bar chart. (**C**) PLS-DA score plots from the healthy (*n* = 63) and rosacea groups (*n* = 57). (**D**) OPLS-DA score plots from the healthy (*n* = 63) and rosacea groups (*n* = 57). (**E**) Visualization of 43 serum differential metabolite expression values in 2 groups by heatmap. *P < 0.05, ***P < 0.001 by 2-tailed unpaired Student’s *t* test. HC, healthy control (blue); Rosacea, rosacea patients (red); SCFAs, short chain fatty acids.

To investigate the potential metabolites involved in the pathogenesis of rosacea, absolute quantitative metabolomics was performed in 57 patients with rosacea before and after treatment with doxycycline of sub-antimicrobial dose and 63 age-, sex-, and BMI-matched healthy controls. Demographic and baseline clinical characteristics of healthy controls and patients with rosacea are presented in Table 1. A total of 306 metabolites were measured by ultra-performance liquid chromatography coupled to tandem mass spectrometry (UPLC-MS/MS), and 183 metabolites were detected, including fatty acids, amino acids, organic acids, carbohydrates, and others (Figure 1A). The difference of relative abundance between the 2 groups was mainly focused on carbohydrates, organic acids, amino acids, fatty acids, and indoles (Figure 1B). Partial least square discriminant analysis (PLS-DA), a supervised multivariate data analysis method, was conducted to evaluate metabolic differences between rosacea (before doxycycline treatment) and healthy control samples. The results clearly distinguished rosacea and healthy control groups, as shown in Figure 1C. Furthermore, orthogonal partial least square discriminant analysis (OPLS-DA) also demonstrated the separation between the 2 groups (Figure 1D), and 43 differential metabolites (including 39 upregulated and 4 downregulated) were identified as potential biomarkers between the patients with rosacea and healthy controls. These differential metabolites were primarily derived from the amino acids (30.2%), fatty acids (27.9%), organic acids (18.6%), carbohydrates (16.3%), carnitines (2.3%), phenols (2.3%), and benzenoids (2.3%) (Figure 1E and Supplemental Table 1; supplemental material available online with this article; https://doi.org/10.1172/jci.insight.161870DS1).

Collectively, these data reveal metabolic profile changes in patients with rosacea, suggesting a state of biochemical metabolic disorder.

### Glutamic acid and aspartic acid are elevated in patients with rosacea.

**Figure 2:**
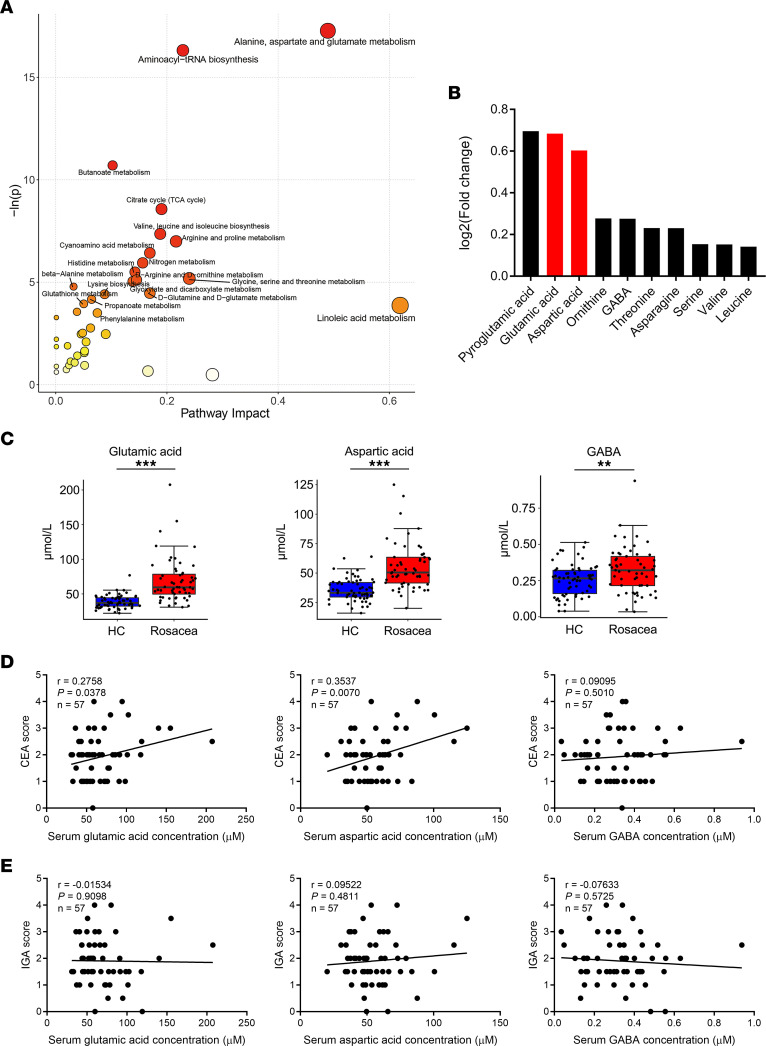
Glutamic acid and aspartic acid are increased in serum of patients with rosacea. (**A**) Pathway analysis bubble plot by hsa set using identified differential metabolites. (**B**) Expression changes of the top 10 differential metabolites in amino acids. (**C**) Box plot of top 3 differential metabolites of alanine, aspartate, and glutamate metabolism pathway. HC, healthy control (blue) (*n* = 63); rosacea, rosacea patients (red) (*n* = 57). Box plots show the interquartile range (box), median (line), and minimum and maximum (whiskers). (**D**) Correlation of serum amino acid levels in patients with rosacea with Clinician’s Erythema Assessment (CEA) scores. (**E**) Correlation of serum amino acid levels in rosacea patients with Investigator’s Global Assessment (IGA) scores. Data represent the mean ± SEM. *P < 0.05, **P < 0.01, ***P < 0.001. Two-tailed unpaired Student’s *t* test or Mann-Whitney *U* test was performed to compare the differences in metabolite levels between the 2 groups (**C**). Spearman’s correlation test was used for the correlation analysis (**D** and **E**).

To explore the functional enrichment of the differential metabolites, pathway analysis was performed by MetaboAnalyst (12). Our results showed that the differential metabolites in patients with rosacea were mainly enriched in pathways related to metabolism and biosynthesis of various amino acids (such as alanine, aspartate, glutamate, arginine, proline, valine, leucine, and so on) and aminoacyl-tRNA biosynthesis, in which the alanine, aspartate, glutamate metabolism pathway was highlighted (Figure 2A). Among all differential amino acids, glutamic acid and aspartic acid were significantly increased in patients with rosacea and ranked as the top 2 amino acids that belonged the to alanine, aspartate, glutamate metabolism pathway (Figure 2, B and C, and Supplemental Figure 1A). To investigate potential clinical implications of these amino acids, we further conducted a correlation analysis, and we found a positive correlation between the Clinician’s Erythema Assessment (CEA) scores and most differential amino acids, especially for glutamic acid and aspartic acid (*r* = 0.2758, *P* = 0.0378; *r* = 0.3537, *P* = 0.0070, respectively) (Figure 2D and Supplemental Figure 1B). However, there was no significant correlation between the Investigator’s Global Assessment (IGA) scores and these amino acids (Figure 2E and Supplemental Figure 1C). To confirm the results about these 2 amino acids, we performed another absolute quantitative metabolomics on 30 patients with rosacea and 20 age-, sex-, and BMI-matched healthy controls recruited from another hospital site to detect the amino acid profile. Similar to our former results, the changes of glutamic acid and aspartic acid were the most notable among other amino acids (Supplemental Figure 1, D and E, and Supplemental Table 2). Likewise, this validation study showed that glutamic acid and aspartic acid were positively correlated with CEA scores, but not IGA scores, which was consistent with our former findings (Supplemental Figure 1, F and G). Collectively, our results indicate that amino acid metabolism, particularly glutamic acid and aspartic acid, may play an important role in rosacea pathogenesis.

### Glutamic acid and aspartic acid supplementation can both aggravate rosacea-like erythema and angiectasis in mice.

**Figure 3:**
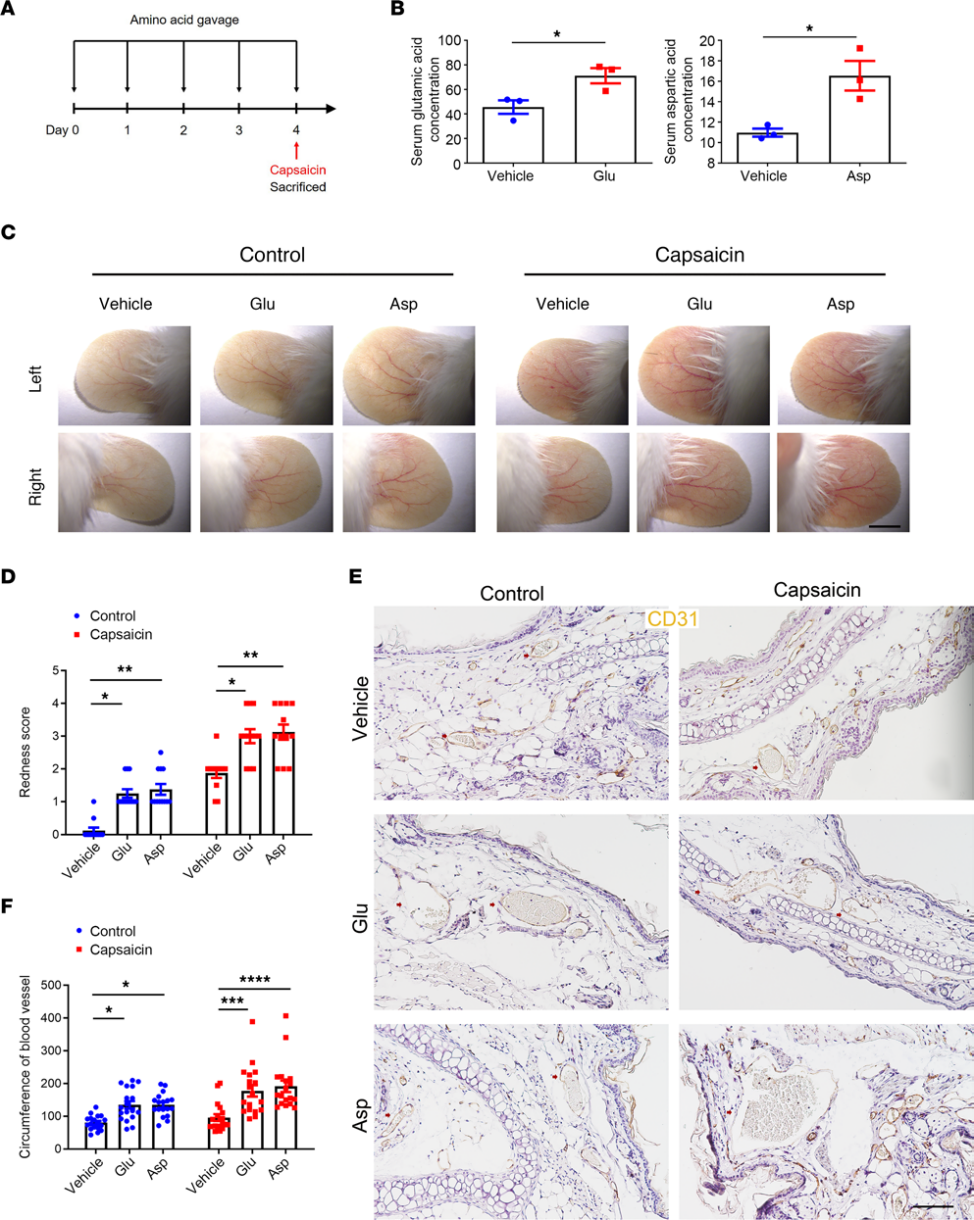
Glutamic acid and aspartic acid exacerbate rosacea-like erythema and vasodilation in mice. (**A**) Schematic diagram of gavage administration of glutamic acid or aspartic acid for 5 continuous days before smearing with capsaicin on ears. Mice were sacrificed on day 4 to conduct subsequent experiments. The mouse experiments were repeated 3 times, and 5–8 mice were included in each group each time. The results of a representative mouse experiment are displayed. (**B**) Serum levels of glutamic acid and aspartic acid in mice after gavage for 5 days continuously (*n* = 3 for each group). (**C**) The ears of mice were smeared with capsaicin or control vehicle (*n* = 6 mice for each group). Images were taken 30 minutes after capsaicin administration. Scale bar, 2 mm. (**D**) The severity of the rosacea-like phenotype was evaluated on account of the redness score (*n* = 6 mice for each group). (**E**) IHC of CD31 on ear sections from mice. Scale bar, 100 μm. (**F**) The circumference of blood vessels in each group was calculated (*n* = 6 mice for each group). All results are representative of at least 3 independent experiments. Data represent the mean ± SEM. *P < 0.05, **P < 0.01, ***P < 0.001, ****P < 0.0001. Two-tailed unpaired Student’s *t* test (**B**) or 1-way ANOVA with Bonferroni’s post hoc test (**D** and **F**) was used.

Erythematotelangiectatic lesions could be considered the initial and essential sign of rosacea and may progress to other clinical manifestations and symptoms (13). It is well known that triggers such as spicy foods are an important dietary inducer for rosacea, and capsaicin is the main active ingredient of spicy foods (14). To further substantiate the potential role of glutamic acid and aspartic acid in rosacea development, 8-week-old BALB/c female mice were administrated with glutamic acid and aspartic acid by gavage for 5 continuous days. On the last day, the mice were smeared with capsaicin on the ear skin (Figure 3A). We first confirmed that glutamic acid and aspartic acid were increased in the serum of gavage-treated mice (Figure 3B). Our results demonstrated that the erythema and vasodilation of ear skin were significantly aggravated in mice with high serum glutamic acid or aspartic acid, and these 2 amino acids could further exacerbate capsaicin-induced erythema and vasodilation (Figure 3, C and D, and Supplemental Figure 2, A and B). By immunohistochemistry of CD31 (a marker of blood vessels), we verified that the circumferences of blood vessels in glutamic acid and aspartic acid groups were both larger than control groups (Figure 3, E and F). To further explore the relationship between inflammation and these 2 amino acids, we conducted histological analysis and real-time quantitative PCR (RT-qPCR). The result showed there was no difference in the infiltration of inflammatory cells in the dermis and the expression of inflammation-related cytokines and chemokines, consistent with our previous findings in the metabolome of patients, where glutamic acid and aspartic acid were not correlated with IGA scores, which represent inflammation levels (Supplemental Figure 2, C–E). We then asked whether other amino acids might have similar effects and treated mice with leucine in the same way. Surprisingly, mice in the leucine group did not present erythema and vasodilation compared with the vehicle group (Supplemental Figure 2, F and G). Moreover, immunohistochemistry of CD31 showed the circumference of blood vessels in the leucine group did not increase compared to the vehicle group (Supplemental Figure 2, H and I), and histological analysis showed leucine had no effect on the inflammatory infiltration of the ear dermis (Supplemental Figure 2, J and K). Overall, these results demonstrate that glutamic acid and aspartic acid can promote the dilation of microvessels and make them more sensitive to irritation.

### Glutamic acid and aspartic acid induce the release of nitric oxide from endothelial cells and keratinocytes.

**Figure 4:**
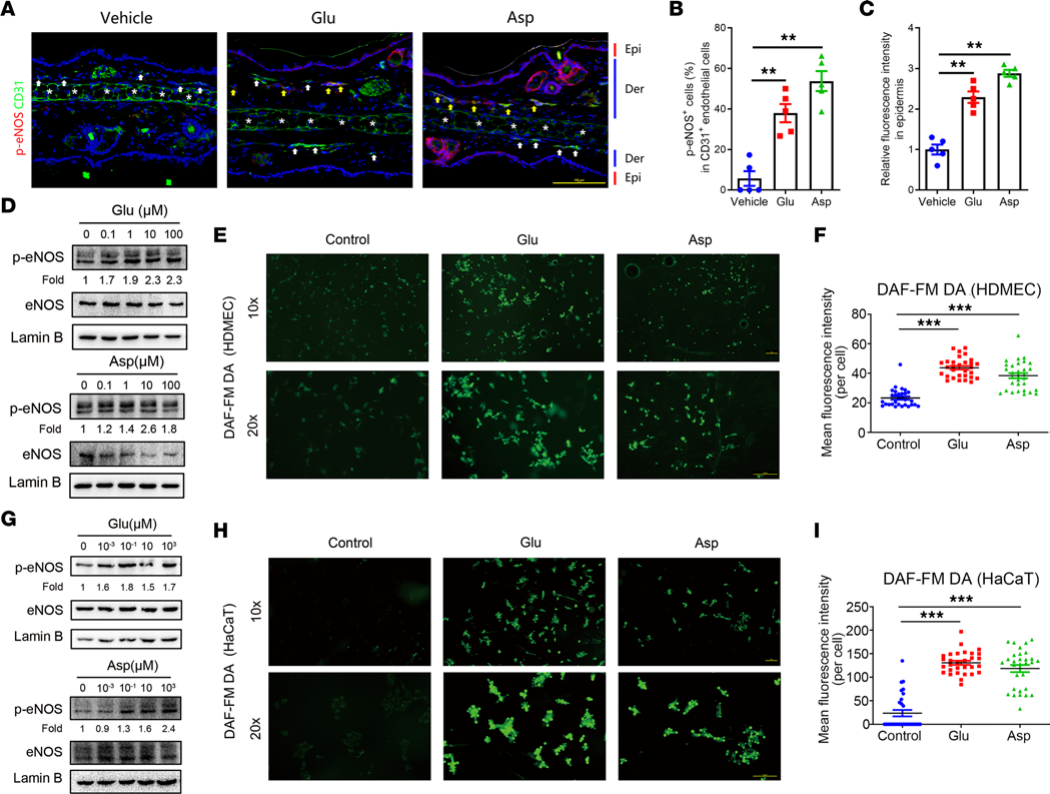
Glutamic acid and aspartic acid stimulate the production of NO from endothelial cells and keratinocytes. (**A**) Representative immunofluorescence images showing CD31 (green) and p-eNOS (red) (CD31, labeling vascular endothelial cells) expression on ear sections from amino acid gavage mice and control group. White arrows indicate the CD31^+^p-eNOS^–^ endothelial cells; yellow arrows indicate the CD31^+^p-eNOS^+^ endothelial cells; white asterisks indicate the ear cartilage with the nonspecific staining or autofluorescence signals. DAPI staining (blue) indicates nuclear localization. Epi, epidermis; Der, dermis. Scale bar, 100 μm. (**B**) Percentage of p-eNOS^+^ endothelial cells (*n* = 5 for each group). (**C**) The quantification of relative fluorescence intensity in epidermis for p-eNOS (*n* = 5 for each group). (**D**) Immunoblot analysis of the p-eNOS and total eNOS in cell lysates from HDMECs after glutamic acid or aspartic acid treatment. Lamin B is the loading control. p-eNOS protein levels were analyzed relative to total eNOS. (**E**) DAF-FM DA staining in HDMEC after glutamic acid or aspartic acid treatment. Scale bar, 100 μm. (**F**) Quantification of intensity of DAF-FM DA fluorescence in HDMECs under the designated treatments (*n* = 30 cells). The quantification results are representative of at least 3 independent experiments. (**G**) Immunoblot analysis of the p-eNOS and total eNOS in cell lysates from HaCaT keratinocytes after glutamic acid or aspartic acid treatment. Lamin B is the loading control. Protein levels of p-eNOS were analyzed relative to total eNOS. (**H**) DAF-FM DA staining in HaCaT keratinocytes after glutamic acid or aspartic acid treatment. Scale bar, 100 μm. (**I**) Quantification of intensity of DAF-FM DA fluorescence in HaCaT keratinocytes under the designated treatments (*n* = 30 cells). The quantification results are representative of at least 3 independent experiments. All results are representative of at least 3 independent experiments. Data represent the mean ± SEM. **P < 0.01, ***P < 0.001. One-way ANOVA with Bonferroni’s post hoc test was used.

Since nitric oxide (NO) is known as the main vasodilative substance, generated in endothelial cells from its precursor l-arginine by the enzymatic action of endothelial NO synthase (eNOS) (15), we wondered whether these amino acids could stimulate NO production. First, by immunostaining of phosphorylated eNOS (p-eNOS), the active form of eNOS (16), we examined the activity of eNOS in skin and found that the percentage of p-eNOS–positive endothelial cells was significantly increased in the skin of mice treated with glutamic acid and aspartic acid (Figure 4, A and B).

To further explore the regulatory function of glutamic acid and aspartic acid in vasodilation, we treated human dermal microvascular endothelial cells (HDMECs) with glutamic acid, aspartic acid, and leucine and found that glutamic acid and aspartic acid increased the phosphorylation of eNOS in a dose-dependent manner (Figure 4D), but leucine did not (Supplemental Figure 3A). To confirm the eNOS activity, NO production was measured using the NO-sensitive DAF-FM DA fluorescence dye in HDMECs. The results showed that glutamic acid or aspartic acid significantly promoted NO production (Figure 4, E and F) while leucine presented no effect (Supplemental Figure 3, B and C). Keratinocytes have been reported to be able to synthesize NO through eNOS (17, 18). Similar to our findings in HDMECs, only the supplementation of glutamic acid or aspartic acid increased the phosphorylation of eNOS and NO production in HaCaT keratinocytes (Figure 4, G–I, and Supplemental Figure 3, D–F). Consistent with in vitro experiments, the activity of eNOS was increased in the epidermis of mouse ears after glutamic acid or aspartic acid treatment (Figure 4, A and C). Collectively, these results support the hypothesis that glutamic acid and aspartic acid facilitate vasodilation possibly by inducing activation of eNOS and increasing NO release.

### Glutamic acid and aspartic acid promote peripheral neurons and keratinocytes to secrete vasodilation-related neuropeptides.

**Figure 5:**
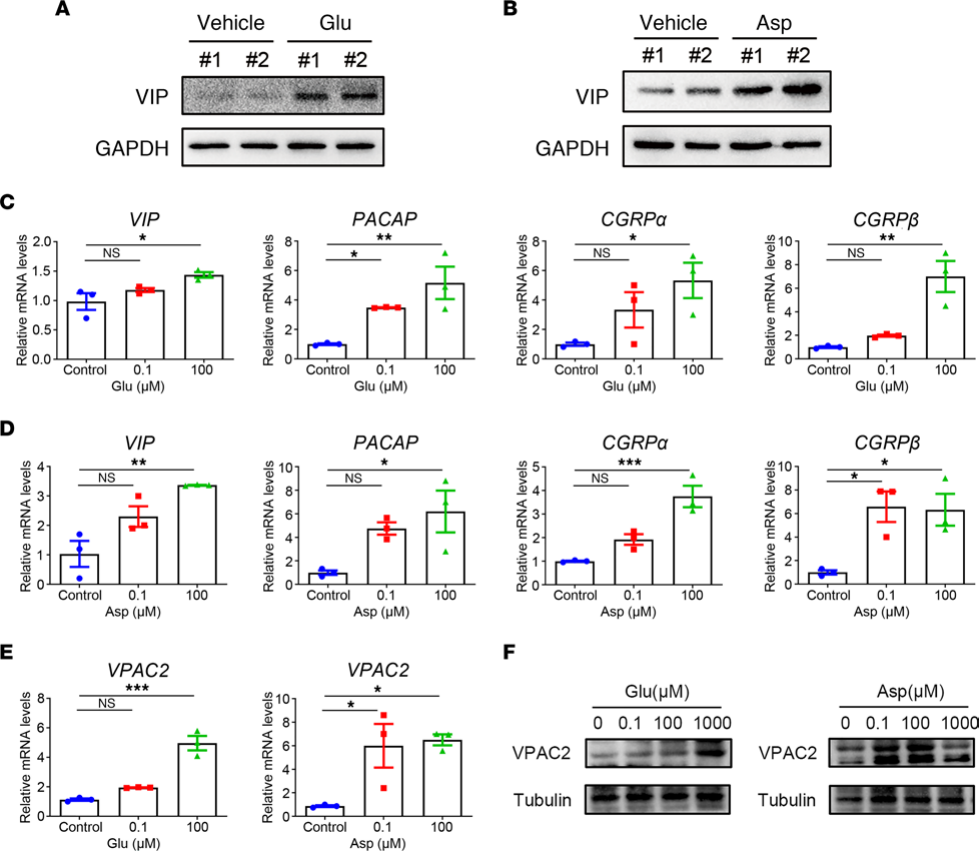
Glutamic acid and aspartic acid induce vasodilation-related neuropeptides from peripheral neurons and keratinocytes. (**A**) Immunoblot analysis of the VIP expression level in DRG neurons from glutamic acid gavage mice and control group. (**B**) Immunoblot analysis of the VIP expression level in DRG neurons from aspartic acid gavage mice and control group. See complete unedited blots in the supplemental material. (**C** and **D**) The mRNA expression levels of *VIP*, *PACAP*, *CGRPα*, and *CGRPβ* in HaCaT keratinocytes after glutamic acid or aspartic acid treatment (*n* = 3 for each group). (**E** and **F**) The mRNA (**E**) and protein (**F**) expression levels of VPAC2 in HDMECs after glutamic acid or aspartic acid treatment (*n* = 3 for each group). All results are representative of at least 3 independent experiments. Data represent the mean ± SEM. *P < 0.05, **P < 0.01, ***P < 0.001. One-way ANOVA with Bonferroni’s post hoc test was used.

Glutamic acid and aspartic acid are major excitatory neurotransmitters of the vertebrate nervous system (19). Vasoactive neuropeptides mainly derived from nerve cells, such as pituitary adenylate cyclase-activating peptide (PACAP) and migraine-associated calcitonin gene-related peptide (CGRP), are upregulated in rosacea skin, and vasoactive intestinal peptide (VIP) receptor–positive cells distribute more densely within the endothelium of patients with rosacea (20–23). Since neuropeptides and their receptors are responsible for local blood flow regulation, they have been considered to induce flushing, a hallmark feature of rosacea, and erythema by neurovascular mechanisms. Therefore, we wondered whether high levels of glutamic acid or aspartic acid could stimulate peripheral neurons to produce neuropeptides. The dorsal root ganglion (DRG) neurons of mice were collected to evaluate the expression of neuropeptides after treatment with glutamic acid or aspartic acid by gavage for 5 days continuously. The results showed that among the investigated neuropeptides, only *VIP* was increased at mRNA levels in DRG neurons of glutamic acid and aspartic acid groups (Supplemental Figure 4A). By immunoblot analysis, we verified that VIP protein levels were also upregulated in DRG neurons of glutamic acid– or aspartic acid–treated mice (Figure 5, A and B). Skin is considered as an important peripheral neuro-endocrine-immune organ closely linked to the central regulatory systems. The local synthesis of neuropeptides also takes place in keratinocytes (24). Thus, we further examined the expression of neuropeptides in keratinocytes, showing that glutamic acid or aspartic acid induced elevation of *VIP*, *PACAP*, *CGRPα*, and *CGRPβ* in HaCaT keratinocytes in a dose-dependent manner (Figure 5, C and D), whereas leucine did not increase the expression of neuropeptides (Supplemental Figure 4C). VIP binds to specific membrane receptor VPACs (including VPAC1 and VPAC2 subtypes). VPACs recognize similarly and bind with high affinity to both PACAP and VIP (25). As expected, we demonstrated that the expression of VPAC2 in HDMECs was increased at mRNA and protein levels, following treatment with glutamic acid or aspartic acid, whereas VPAC1 was unaffected (Figure 5, E and F, and Supplemental Figure 4B). In addition, leucine had no effect on the expression of either *VPAC1* or *VPAC2* (Supplemental Figure 4D).

These results suggest that circulating elevated glutamic acid and aspartic acid may contribute to the aggravation of rosacea erythema by promoting secretion of vasodilation-related neuropeptides from peripheral neurons and keratinocytes.

### Serum glutamic acid and aspartic acid decrease after doxycycline treatment in patients with rosacea.

**Figure 6:**
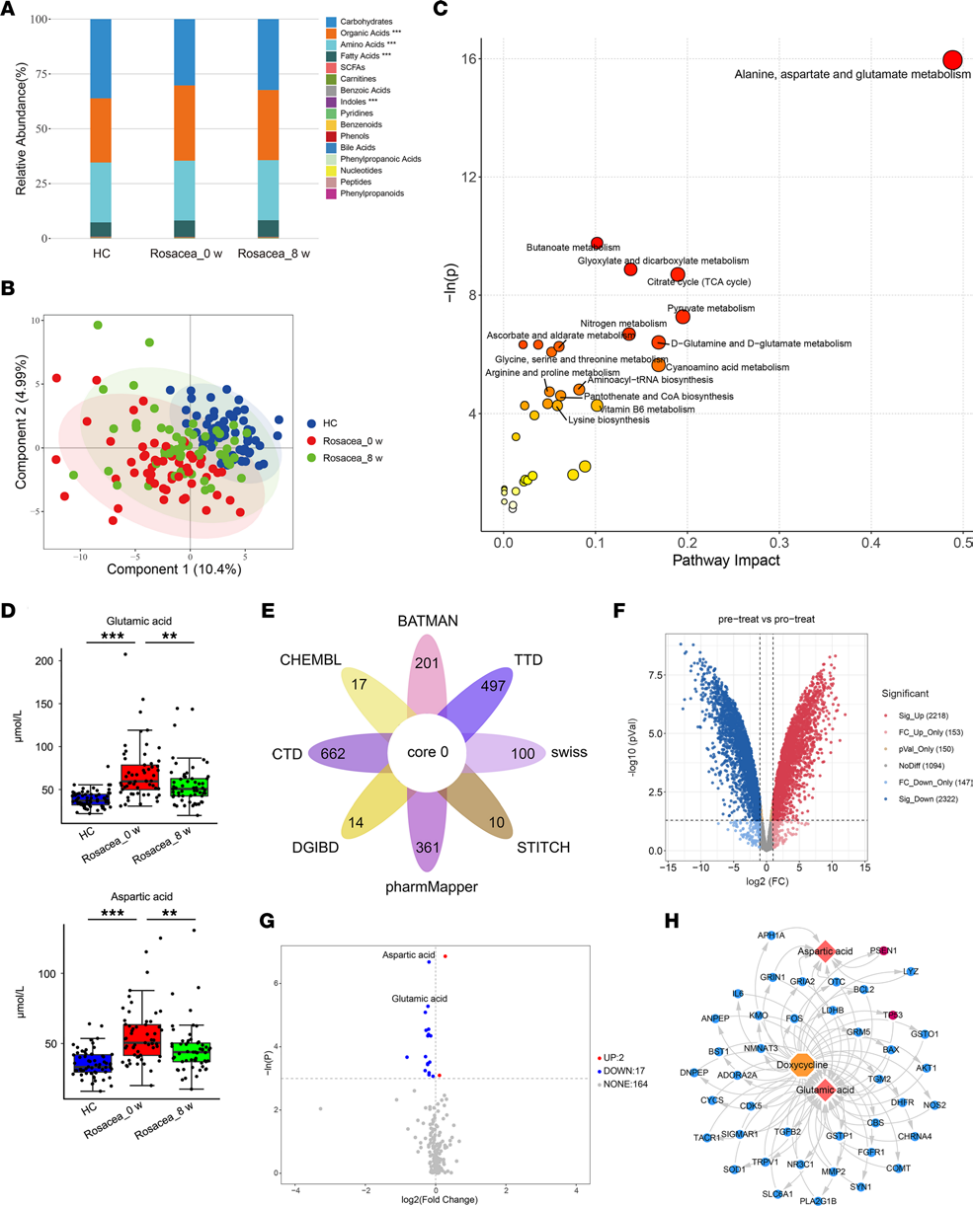
Glutamic acid and aspartic acid decrease in serum after doxycycline treatment in patients with rosacea. (**A**) The relative abundance of each metabolite classes in different groups is shown in the stacked bar chart. (**B**) PLS-DA score plots from healthy control (*n* = 63) and rosacea group before medication and rosacea group after treatment (*n* = 57). (**C**) Pathway analysis bubble plot by hsa set using identified differential metabolites between rosacea group before medication and rosacea group after treatment. (**D**) Box plot of serum glutamic acid and aspartic acid levels in different groups. Box plots show the interquartile range (box), median (line), and minimum and maximum (whiskers). (**E**) Venn diagram of possible targets of doxycycline. (**F**) Volcano plot of differentially expressed genes from the transcriptome of the liver from doxycycline-treated and vehicle control–treated rats. (**G**) Volcano plot of differential metabolites of patients with rosacea before and after doxycycline administration. (**H**) Network diagram of multiomics analysis of network pharmacology, transcriptomics, and metabolomics. Data represent the mean ± SEM.**P < 0.01, ***P < 0.001. One-way ANOVA with Bonferroni’s post hoc test was used. HC, healthy control (blue); Rosacea_0 w, rosacea patients before medication (red); Rosacea_8 w, rosacea patients after 8-week of doxycycline treatment (green).

Since glutamic acid and aspartic acid may contribute to the development and exacerbation of rosacea, we then wondered whether clinical therapies improve the symptoms of disease through these amino acids. Sub-antimicrobial dose of doxycycline is the only FDA-approved oral medication for rosacea (26). Therefore, we also detected the serum metabolites of the same 57 patients after oral doxycycline (40 mg/d) for 8 weeks. The clinical outcomes showed that their conditions improved markedly after doxycycline treatment (Table 2). Consistent with the clinical manifestations in patients, the difference of metabolic profiles between the rosacea group after treatment and healthy controls was attenuated compared with the rosacea group before medication (Figure 6, A and B), and the changes are mainly accumulated in amino acids, carbohydrates, organic acids, and fatty acids (Supplemental Table 3). Pathway analysis for differentially expressed metabolites highlighted the enrichment of alanine, aspartate, and glutamate metabolism pathway again when comparing the rosacea group after treatment with that before medication (Figure 6C). The concentrations of glutamic acid and aspartic acid were markedly rescued after 8 weeks’ continuous doxycycline treatment, though the abundance of these metabolites was still a little higher compared with healthy controls (Figure 6D). However, other amino acids displayed few or no changes, and there was no significant correlation between the serum levels of most amino acids (including glutamic acid and aspartic acid) and disease severity in patients after doxycycline treatment (Supplemental Figure 5). These data suggested the possibility that doxycycline might improve rosacea symptoms via targeting glutamic acid and aspartic acid. To verify this possibility, we combined network pharmacology, metabolomics before and after doxycycline treatment, and transcriptomic analysis of liver in rats administered with doxycycline or vehicle. Specifically, we first predicted the targets of doxycycline by database query and obtained 928 genes (Figure 6E). The transcriptome of liver from doxycycline-treated and vehicle control–treated rats revealed 3,224 differentially expressed genes (Figure 6F). After integrating genes’ names from different species, 347 genes overlapped the 928 doxycycline-targeted genes and the 3,224 liver transcriptome genes. A total of 19 differential metabolites were screened by unidimensional analysis in patients with rosacea after 8 weeks of doxycycline treatment, compared with baseline. Among them, glutamic acid and aspartic acid had prominent reductions (Figure 6G). Then, multiomics analysis of network pharmacology, transcriptomics, and metabolomics was performed for 19 differentially expressed metabolites in serum and these 347 genes, and we found a total of 41 genes for glutamic acid and aspartic acid (38 genes for glutamic acid, 8 genes for aspartic acid, and 5 for both) (Supplemental Table 4). Among these genes, tumor protein p53 (TP53) and presenilin 1 (PSEN1) have been reported to affect the synthesis of these 2 amino acids. Downregulation of TP53 after doxycycline treatment may inhibit the release of glutamic acid synthesized by hepatocytes into the peripheral circulation, thereby reducing the serum glutamic acid concentration after treatment (27). Mutations in PSEN1 reduce the aspartic acid synthesis derived from leucine metabolism (28). Therefore, TP53 and PSEN1 were highlighted as the critical factors by which doxycycline may regulate glutamic acid or aspartic acid in patients with rosacea (Figure 6H). These data suggest that doxycycline can alleviate the rosacea symptoms possibly via targeting the amino acid metabolic pathway.

In conclusion, we reveal that aberrant amino acid metabolism promotes neurovascular reactivity in the development of rosacea and suggest targeting dysregulated metabolism might be a novel therapy for rosacea.

## Discussion

A systemic review and meta-analysis study have put forward the possibility that rosacea may have linkage with hypertension, dyslipidemia, and metabolic syndrome (29). Another clinical study revealed that rosacea patients were associated with higher uric acid levels and BMI values (30). However, direct evidence on the relationship between rosacea and metabolic disorder is still lacking. Here, we recruited 63 healthy controls and 57 patients with rosacea, investigated their metabolome changes in serum, and revealed significantly altered metabolic signatures in rosacea, especially for amino acid–related metabolic pathways.

Glutamic acid and aspartic acid are members of acidic amino acids and were found elevated in the serum of patients with type 1 diabetes or psoriasis (31, 32). Previous studies also reported glutamic acid induced [Ca^2+^]*_i_* oscillations in astrocytes, thus dilating arterioles, which may explain the mechanism of hyperemia (33). Knockdown of glutamine synthetase in endothelial cells, which converts glutamate to glutamine, suppressed ocular angiogenesis (34). Here, we revealed the enrichment of the aspartate and glutamate metabolism pathway and the accumulation of glutamic acid and aspartic acid in serum samples of patients with rosacea. Based on our results that the concentration of these 2 amino acids was positively associated with erythema score, we hypothesized that glutamic acid and aspartic acid promote the progression of rosacea by regulating vascular function. In line with our hypothesis, supplementation of glutamic acid or aspartic acid aggravated the erythema and vasodilation of microvessels, with or without the irritation of capsaicin.

In searching for the potential mechanisms by which glutamic acid or aspartic acid promotes rosacea-like angiectasis, we focused on endothelium-dependent vasodilation. NO is a major endogenous local regulator of vascular tone, and endothelium-derived NO is generated mainly by eNOS. The enzyme can be activated in both calcium-dependent and posttranslational modificatory ways, for example, phosphorylation at Ser1177 (15). Recent studies have found that glutamate can induce endothelium-derived NO release and lead to vasodilation of arterioles and capillaries in the brain (35, 36). In the present study, we demonstrated that the expression of p-eNOS in ear vessels of mice was increased after gavage of glutamic acid or aspartic acid. Moreover, glutamic acid and aspartic acid can activate eNOS and increase NO release in HDMECs and HaCaT keratinocytes. From here we see that apart from the autocrine function of HDMECs, keratinocytes also act on neighboring HDMECs in a paracrine way to promote the development of rosacea via NO.

Glutamic acid and aspartic acid are main excitatory neurotransmitters, and their metabolic disturbance is mainly related to the dysfunction of the central nervous system (37–39). They also participate in neurovascular coupling such that neuronal activation increases blood flow (40). However, the effects of these 2 amino acids from the circulation on the peripheral nervous system remain relatively unclear. In the present study, we found that elevated glutamic acid and aspartic acid in circulation can stimulate the secretion of vasodilation-related neuropeptides by peripheral neurons, such as VIP. Previous studies reported VIP and PACAP could regulate glutamate metabolism, but hardly any research focused on the effect amino acids had on VIP in peripheral nerves (41). We first found the stimulation of glutamic acid and aspartic acid could increase the expression of VIP in mouse DRG neurons. VIP, a peptide of the secretin/glucagon family with 28 amino acids, was identified not only in the gastrointestinal tract but also in the central nervous system and peripheral nerves. As a neuropeptide, VIP participates in a variety of biological activities, including vasodilation. VIP could dilate coronary arteries, meningeal vessels, pulmonary arteries, as well as cutaneous microvessels (42–46). It is reported that expression of the receptor of VIP is positive in slice biopsies from patients with rosacea compared with controls (22). We speculated that the expression of VIP induced by amino acids could be an important cause of neurogenic vasodilation in rosacea. In addition, evidence for a neuropeptidergic system in skin keratinocytes was reported (24). Here, we demonstrated that *VIP*, *PACAP*, *CGRPα*, and *CGRPβ*, mediating angiogenesis and vasodilation in skin, were all upregulated in keratinocytes by glutamic acid and aspartic acid. Collectively, these data suggest that glutamic acid and aspartic acid are involved in vascular regulation of rosacea by directly promoting endothelium-dependent vasodilation and indirectly inducing the secretion of vasodilation-related neuropeptides.

Apart from neurovascular dysregulation, an inflammatory reaction also plays a vital role in rosacea pathogenesis. In our study, we evaluated the relationship between amino acids and IGA scores, and the results showed there was no correlation between most amino acids and IGA scores. Moreover, we revealed glutamic acids and aspartic acids failed to increase the infiltration of inflammatory cells in mouse ears as well as related cytokines and chemokines. Therefore, we speculated that these amino acids may not directly cause inflammation. It is reported that neuropeptides like PACAP and CGRP stimulate T cells, macrophages, and mast cells to initiate or amplify the inflammatory response, which is called neurogenetic inflammation (47). Therefore, we may generate the hypothesis that amino acids affect the neurovascular functions first, by inducing the release of nitric oxide and vasodilation-related neuropeptides, which may further aggravate inflammatory reaction if stimulated by other factors.

Doxycycline 40 mg, the only oral medicine approved by the FDA for the treatment of rosacea, was reported to significantly reduce the severity of disease as measured by erythema score and flushing episodes (48). In the present study, we found the increase of glutamic acid and aspartic acid could be reversed by doxycycline treatment in patients with rosacea, and CEA scores were also improved after treatment. We speculated doxycycline improves neurovascular sensitivity by reducing glutamic acid and aspartic acid levels. However, it is reported that low-dose doxycycline could decrease systemic inflammation (49–51). Our study found IGA score also declined after treatment, and there was no correlation with the above amino acids. Hence, we hypothesized that, in addition to reducing neurovascular sensitivity by reducing the levels of glutamic acid and aspartic acid, doxycycline may also improve disease by antiinflammatory effects in patients treated with doxycycline. Considering the evidence above, we wanted to further explore how doxycycline regulates the metabolism of glutamic acid and aspartic acid. Through integration analysis of network pharmacology, metabolomics before and after doxycycline treatment, and transcriptomic analysis of liver tissue from doxycycline-treated and vehicle control–treated rats, we emphasized the critical role of glutamic acid and aspartic acid in the pathogenesis of rosacea and determined doxycycline-targeting gene TP53 was involved in glutamate metabolism, while PSEN1 was involved in aspartic acid metabolism. TP53, located on the short arm of chromosome 17, is critical in tumor suppression. The activation of wild-type P53 increases the expression of cystine/glutamate antiporter xCT to export intracellular glutamic acid (27). The downregulation of TP53 after doxycycline treatment may suppress the release of glutamic acid synthesized in hepatocytes into peripheral circulation, thus reducing the concentration of serum glutamic acid in patients after treatment. PSEN1, belonging to the aspartyl proteases family, is generally recognized as being associated with Alzheimer’s disease. It is reported that the mutation of PSEN1 in astrocytes reduces the aspartic acid synthesis derived from leucine metabolism (28). Consistently, the expression of PSEN1 decreased after doxycycline treatment, as does the serum level of aspartic acid. Based on these findings, we speculated that doxycycline may suppress glutamate secretion by reducing the expression of TP53 and aspartate synthesis by reducing PSEN1, thereby alleviating the symptoms of rosacea.

Admittedly, in view of the fact that rosacea mostly occurs in young and middle-aged women, the 2 cohorts we recruited were both composed of women, which does not fully reflect reality. We are trying to collect more evidence to confirm whether our findings apply to men.

In conclusion, we reveal that aberrant amino acid metabolism promotes neurovascular reactivity in the development of rosacea and suggest targeting dysregulated metabolism might be a novel therapy for rosacea.

## Methods

### Patient information and sample collection.

A total of 57 rosacea patients before and after treatment with doxycycline of sub-antimicrobial dose and 63 age-, sex-, and BMI-matched healthy controls were recruited from the Department of Dermatology in Xiangya Hospital, Central South University. As for the validation population, 30 patients diagnosed with rosacea and 20 age-, sex-, and BMI-matched healthy controls were recruited from the Department of Dermatology in the First Hospital of Changsha. Inclusion criteria included newly diagnosed and untreated rosacea patients according to the 2017 diagnostic criteria determined by the National Rosacea Society Expert Committee without any other metabolic comorbidities (52). The exclusion criteria for all the participants were systemic diseases and other skin disease, history of systemic immunomodulators or antibiotics, intake of prebiotics or probiotics, extreme diets in the previous 12 weeks, pregnancy, and lactation. Patients were clinically evaluated for IGA scores and CEA scores as previously described (53). Demographic and baseline clinical characteristics of 57 rosacea patients and 63 healthy controls are listed in Table 1. Serum samples of participants were collected at initial diagnosis and follow-up 8 weeks after oral doxycycline (40 mg/d) treatment. Peripheral blood samples were collected after overnight fasting for 10 hours and set aside for 30 minutes to obtain serum. All samples were stored at –80°C immediately after collection until analysis.

### Targeted metabolomics sequencing and analysis.

The metabonomics analysis was performed by Q300 Kit (Metabo-Profile) as previously described (11, 54). Specifically, metabolites were quantitated using a UPLC-MS/MS system (ACQUITY UPLC-Xevo TQ-S, Waters Corp.). All of the standards were obtained from MilliporeSigma, TRC Chemicals, and Steraloids Inc. To obtain individual stock solution, we weighed and prepared standards in water, methanol, hydrochloric acid solution, or sodium hydroxide solution. Each stock solution was appropriately mixed to create stock calibration solutions. A total of 25 μL of ice-bath serum was added to a 96-well plate. A total of 120 μL of ice-cold methanol with partial internal standards was added to each sample and then vortexed vigorously for 5 minutes. The plate was centrifuged at 4,000*g* for 30 minutes at 10°C, 30 μL of supernatant was transferred to a clean 96-well plate, and then 20 μL of derivative reagents was added to each well. The plate was sealed and the derivatization was carried out for 60 minutes at 30°C. Next, samples were diluted by 330 μL of ice-cold 50% methanol solution. Then the plate was stored at –20°C for 20 minutes and centrifuged at 4,000*g* at 4°C for 30 minutes. After transferring 135 μL of supernatant to a new 96-well plate containing 10 μL internal standards in each well, we added serial dilutions of derivatized stock standards to the left wells. Finally, the sealed plate was prepared for LC-MS analysis. The UPLC instrument settings were as follows: column: ACQUITY UPLC BEH C18 1.7 μM; column temperature: 40°C; sample manager temperature: 10°C; mobile phases: water with 0.1% formic acid (A) and acetonitrile (70:30, B); gradient conditions: 0–1 minutes (5% B), 1–11 minutes (5%–78% B), 11–13.5 minutes (78%–95% B), 13.5–14 minutes (95%–100% B), 14–16 minutes (100% B), 16–16.1 minutes (100%–5% B), 16.1–18 minutes (5% B); flow rate: 0.40 (mL/min); injection volume: 5.0 μL. The conditions of mass spectrometer were as follows: capillary: 1.5 kV and 2.0 kV; source temperature: 150°C; desolvation temperature: 550°C; desolvation gas flow: 1,000 L/h.

The acquired raw data were assessed by the MassLynx software (v4.1, Waters Corp.) to perform peak integration, quantitation, and calibration for each metabolite. Statistical analysis was processed by the powerful package from R studio. The concentration of substances was determined by making the comparison between the unknown sample and the calibration curve. Then, the iMAP software (version 1.0; Metabo-Profile) was operated for the targeted metabolites. Metabolites were identified based on the Human Metabolome Database and the Kyoto Encyclopedia of Genes and Genomes (KEGG). To understand the difference of metabolomics profiles between patients with rosacea and healthy people, multivariate statistical analyses, including PLS-DA and OPLS-DA, were carried out. Meanwhile, univariate statistical analyses, such as 2-tailed Student’s *t* test, Mann-Whitney-Wilcoxon (*U* test), 1-way ANOVA, Kruskal-Wallis, and correlation analysis, were used to identify the altered metabolites in patients with rosacea. KEGG pathway enrichment analysis was conducted using the KEGG database (version 89.1) with hypergeometric test comparing all the identified metabolites. The metabolites with variable importance on projection > 1 and *P* < 0.05 were considered significantly changed metabolites.

### Mice and treatments.

Age- and sex-matched BALB/c mice were purchased from Hunan SLAC Laboratory Animal Co., Ltd. All mice were bred and maintained under specific pathogen–free conditions with food and water ad libitum and were acclimatized to the new environment for 1 week before experiments. Eight-week-old BALB/c mice were treated with glutamic acid, aspartic acid, or leucine (MilliporeSigma) at a dosage of 2 mg/kg or 10 mg/kg per day from day 1 to day 5 by gastric perfusion. Mice from control group were treated with the same amount of normal saline. Capsaicin or vehicle was applied topically to the ears of the mice on day 5. The mice were imaged and euthanized within 30 minutes after topical application for ear lesion and subsequent analysis. Ear biopsy specimens were for histological analysis, immunohistochemistry, immunofluorescence, and quantitative PCR (qPCR); serum samples were for quantification of amino acids; DRG biopsy specimens were for qPCR and immunoblotting.

### Quantification of amino acids of mouse serum samples.

Peripheral blood samples of mice were centrifuged to obtain serum samples. We mixed 100 μL aliquots with 400 μL of cold methanol/acetonitrile (1:1, v/v) to remove the protein. The mixture was centrifuged for 20 minutes (14,000*g*, 4°C). The supernatant was dried in a vacuum centrifuge. For LC-MS analysis, the samples were redissolved in 100 μL acetonitrile/water (1:1, v/v) and adequately vortexed, then centrifuged (14,000*g*, 4°C, 15 minutes). The supernatants were collected for LC-MS/MS analysis. In electron spray ionization–positive modes, the conditions were set as follows: source temperature 500°C, ion source gas 1 (Gas1): 40, ion source gas 2 (Gas2): 40, curtain gas: 30, ion sapary voltage floating 5,500 V; adopt the multiple reaction monitoring mode detection ion pair. The Multiquant software was used to extract chromatographic peak area and retention time. The metabolites were identified by AA standards after retention time correction. All amino compound standards were purchased from MilliporeSigma.

### Histological analysis.

The histological analysis was carried out as per a previous study (55, 56). Formalin-fixed and paraffin-embedded ear skins of mice were cut into 5 μm skin sections and then stained with H&E. To determine the histological features, the number of infiltrating cells in the dermis was averaged in 6 randomly selected microscopic areas (original magnification, 200×) in each mouse.

### Immunohistochemistry.

Ear samples of mice were fixed in formalin and then embedded in paraffin and were cut into 5 μm skin sections. Immunohistochemistry was performed according to previous methods (57). Skin sections were incubated in primary antibody CD31 (1:100, Cell Signaling Technology, 77699s). The averaged circumference of primary capillaries stained by CD31 in 3 random hpfs for each mouse (*n* = 6) in each group was measured using ImageJ.

### Immunofluorescence.

Immunofluorescence of skin sections was conducted as previously described (58). Briefly, ear skins from mice were fixed in 4% paraformaldehyde and then frozen in OCT. Ear sections were cut into 8 μm, then washed with phosphate-buffered saline (PBS) 3 times. After being blocked for 60 minutes with blocking buffer (5% normal donkey serum, 1% BSA, 0.3% Triton X-100), sections were incubated in primary antibodies CD31 for immunofluorescence (1:100, BD Biosciences, 558736) and p-eNOS (1:100, Cell Signaling Technology, 9571s) overnight at 4°C. After wash, we added Alexa Fluor 488– or 594–conjugated secondary antibody (1:500, Thermo Fisher Scientific, A21208 and A21207, respectively) on sections for 60 minutes at room temperature. Next, we washed sections with PBS and counterstained them with DAPI. All pictures were acquired with an ECLIPSE Ni-U Microscope. The number of positive cells was counted and averaged in 5 randomly selected microscopic fields (original magnification, 200×) in each mouse.

### Cell culture and treatment.

HaCaT keratinocytes (immortalized human keratinocyte cell line), obtained from NTCC (Biovector Science Lab), were cultured in DMEM (Gibco, Thermo Fisher Scientific) supplemented with 10% fetal bovine serum, 1% penicillin-streptomycin (Thermo Fisher Scientific), and 1% glutamine (Invitrogen) at 37°C in a humidified CO_2_ incubator (5% CO_2_). HDMEC line purchased from ATCC was cultured in MCB131 (Gibco, Thermo Fisher Scientific) supplemented with 10% fetal bovine serum, 1% penicillin-streptomycin, 10 ng/mL human epidermal growth factor, 400 ng/mL hydrocortisone, and 1% glutamine at 37°C in a humidified CO_2_ incubator (5% CO_2_). For amino acid treatment, at a confluence of 50%, cells were starved overnight, then incubated with glutamic acid, aspartic acid, or leucine (at indicated doses) for the indicated time. All experiments were performed at least 3 times.

### RT-qPCR.

Total RNA was extracted from mouse ears, DRG neurons, HaCaT keratinocytes, and HDMECs by TRIzol reagent (Thermo Fisher Scientific), and a NanoDrop spectrophotometer (ND-2000, Thermo Fisher Scientific) was employed for RNA quality control. mRNA was reverse-transcribed into cDNA by the Maxima H Minus First Strand cDNA Synthesis Kit with dsDNase (Thermo Fisher Scientific) according to the manufacturer’s instructions. The real-time PCR was conducted with iTaq Universal SYBR Green Supermix (Bio-Rad) on a LightCycler 96 (Roche) thermocycler. The relative expression of each gene relative to GAPDH was analyzed by using the ΔCT method, and the fold change was normalized to the control group. The primer sequences of genes used in this study are listed in Supplemental Table 5.

### Immunoblotting.

The mouse DRG biopsies and cells, washed with cold PBS, were lysed with RIPA buffer including protease inhibitors (Thermo Fisher Scientific). We took the supernatant after centrifugation (12,000*g*, 15 minutes, 4°C). The proteins acquired were quantified through bicinchoninic acid assay (Thermo Fisher Scientific) and separated on SDS-PAGE and transferred to a PVDF membrane. The membrane was then blocked with 5% nonfat milk for 1 hour at room temperature and probed with primary antibodies overnight at 4°C. We washed the membrane 3 times in TBS containing 0.1% Tween 20 and incubated with secondary antibodies HRP-conjugated Goat anti-Mouse IgG (1:10,000, Santa Cruz Biotechnology, sc-2005) and HRP-conjugated Goat anti-Rabbit IgG secondary antibody (1:10,000, Santa Cruz Biotechnology, sc-2004) for 1 hour at room temperature. The immunoreactive bands were revealed by the HRP substrate (Luminata, MilliporeSigma) on ChemiDoc XRS+ system (Bio-Rad). GE Healthcare (now Cytiva) ImageQuant LAS 4000 Mini was used for data analysis. The primary antibodies in this study were as follows: Rabbit anti–p-eNOS (phospho S1177, 1:1,000, Cell Signaling Technology, 9571s), Rabbit anti-eNOS (1:1,000, Cell Signaling Technology, 9572s), Rabbit anti-VPAC2 (1:500, AiFang biological, AF06401), Rabbit anti-VIP (1:500, Santa Cruz Biotechnology, sc-25347), Rabbit anti–Lamin B (1:2,000, Abcam, ab16048), Mouse anti–α Tubulin (1:5,000, Abcam, ab7291), Mouse anti-GAPDH (1:10,000, Proteintech, 60004-1), and Mouse anti-β Actin (1:2,000, Santa Cruz Biotechnology, sc-47778).

### Measurement of intracellular NO production.

The production of NO level in HaCaT keratinocytes and HDMECs was measured using a fluorescent indicator, DAF-FM DA. At a confluence of 50%, we treated cells with amino acid for 1 hour and removed the supplement, then loaded with DAF-FM DA for 20 minutes at 37°C. Thereafter, cells were gently washed 3 times with PBS to remove extracellular DAF-FM DA. All pictures were acquired with an ECLIPSE Ni-U Microscope, and the average fluorescence intensity of 30 cells in each repeated experiment was measured with ImageJ (NIH).

### Multiomics analysis of network pharmacology, transcriptomics, and metabolomics.

The possible target genes of doxycycline were predicted based on BATMAN-TCM database, ChEMBL database, Comparative Toxicogenomics Database, DGIBD database, PharmMapper database, Swiss Target Prediction database, STITCH database, and Therapeutic Target Database, then by taking the intersection of all genes across databases. Next, the transcriptome of rat liver was analyzed by the DESeq package of R studio to obtain differentially expressed genes using liver sequencing data from rats with doxycycline or vehicle treatment deposited at the Gene Expression Omnibus database, under accession number GSE59923 (https://www.ncbi.nlm.nih.gov/geo/). After making genes’ names uniform across species, these molecular data were intersected with doxycycline-targeted genes. All the raw data of metabolites were standardized through normalization by sum, square root transformation, and Pareto scaling. The univariate statistical analyses were carried out to identify the altered metabolites in patients with rosacea before and after doxycycline treatment. Target genes by which doxycycline regulates glutamic acid and aspartic acid in rosacea patients were identified by metabolome-transcriptome combined analysis of varied metabolites and genes using MetaboAnalyst software (version 5.0).

### Statistics.

All statistical analyses were carried out using GraphPad Prism 6 (GraphPad Software). The normal distribution and similar variance of data from different groups were examined. The significance of differences (*P < 0.05, **P < 0.01) between groups was determined by 2-tailed unpaired Student’s *t* test for 2 groups’ comparisons or 1-way ANOVA with Bonferroni’s post hoc test for comparisons between multiple groups. When the data were not normally distributed or there existed heterogeneity variances between the 2 groups, statistical significance between different groups was determined by Whitney-Wilcoxon (*U* test) and Kruskal-Wallis. We performed Pearson’s *r* test or Spearman’s *r* test (for abnormally distributed data) for correlation analysis. All data represent the mean ± SEM.

### Study approval.

All human studies were approved by the ethical committee of the Xiangya Hospital, Central South University, and written informed consent was obtained from all participants. All mice were housed in specific pathogen–free conditions, and all procedures were conducted according to the instructions and permissions of the ethical committee of the Xiangya Hospital, Central South University.

## Author contributions

TL, WX, and ZD performed most of the experiments, analyzed the data, and wrote the manuscript. MC assisted with the establishment of mouse models. MC and SX assisted with molecular cloning. WX, TL, and ZZ collected the clinical samples. QP assisted with immunohistochemistry experiments. RM performed network pharmacology, metabolomics, and transcriptomic combined analysis. QW and HX provided technical support and suggestions for the project. JL and ZD conceived the project and supervised the study. ZD, JL, WX, and TL designed the experiments, analyzed and interpreted data, and wrote the manuscript.

## Supplementary Material

Supplemental data

## Figures and Tables

**Table 1:**
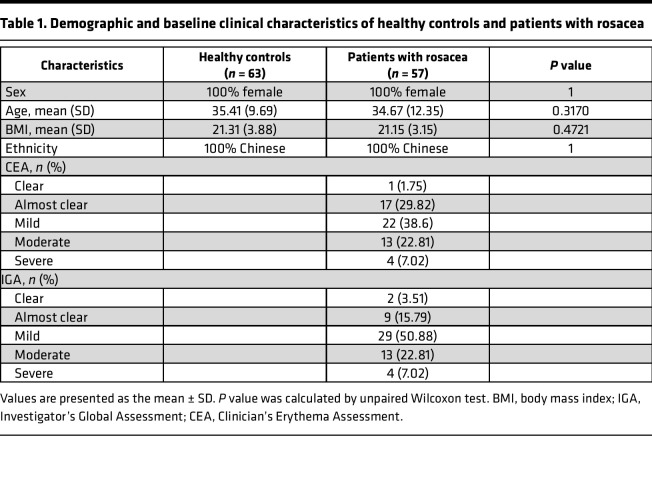
Demographic and baseline clinical characteristics of healthy controls and patients with rosacea

**Table 2:**
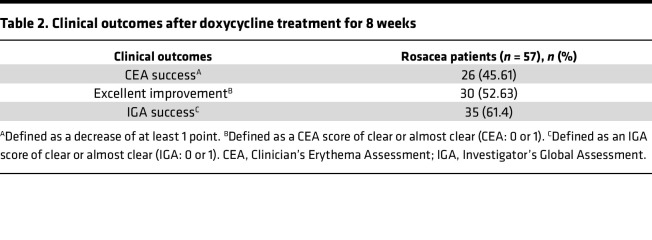
Clinical outcomes after doxycycline treatment for 8 weeks

## Version 1 10/11/2022

In-Press Preview

## Version 2 11/22/2022

Electronic publication
