# Supplementary material for: Aberrant amino acid metabolism promotes neurovascular reactivity in rosacea
Source: JCI Insight. 2022 Nov 22;7(22):e161870. doi: 10.1172/jci.insight.161870 (PMC9746811; doi:10.1172/jci.insight.161870)
Supplement: Supplemental data [file jciinsight-7-161870-s044.pdf]

Supplemental figures and figure legends

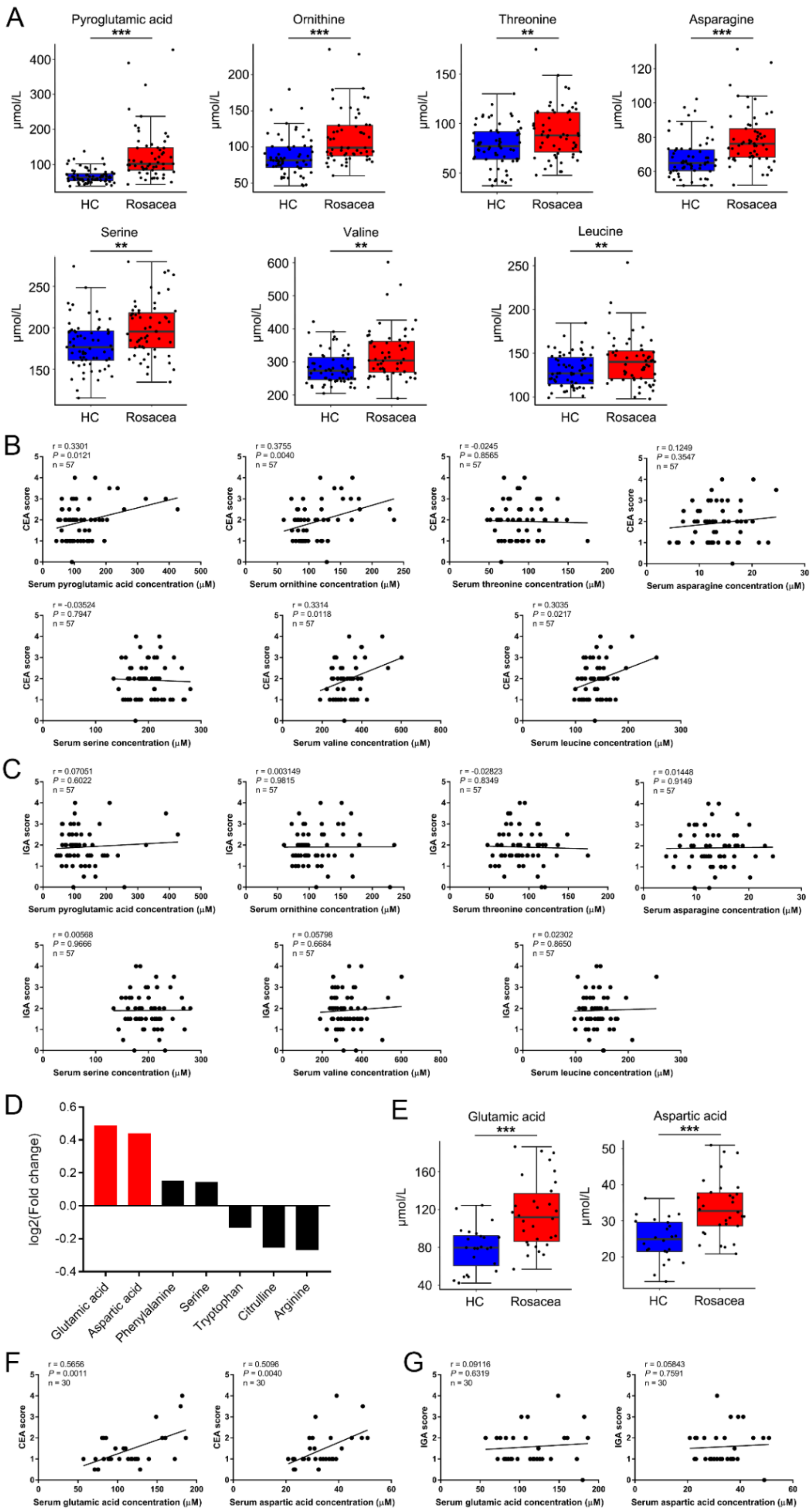

Supplemental Figure 1. Expression changes of amino acids in serum of rosacea patients.

(**A**) Boxplot of top 10 differential amino acids except glutamic acid, aspartic acid and GABA. HC, healthy control (blue) (n=63); Rosacea, rosacea patients (red) (n=57). (**B**) Correlation of serum amino acid levels in rosacea patients with Clinician's Erythema Assessment (CEA) scores. (**C**) Correlation of serum amino acid levels in rosacea patients with Investigator's Global Assessment (IGA) scores. (**D**) Expression changes of the top 7 differential metabolites in amino acids in the validation cohort. (**E**) Boxplot of glutamic acid and aspartic acid in the validation cohort. HC, healthy control (blue) (n=20); Rosacea, rosacea patients (red) (n=30). (**F**) Correlation of glutamic acid and aspartic acid levels in rosacea patients in the validation cohort with CEA scores. (**G**) Correlation of serum amino acid levels in rosacea patients in the validation cohort with IGA scores. Data represent the mean ± SEM. $^*P < 0.05$, $^{**}P < 0.01$, $^{***}P < 0.001$. 2-tailed unpaired Student's *t*-test or Mann-Whitney *U* test was performed to compare the differences in metabolite levels between the 2 groups (A and E). Spearman's correlation test was used for the correlation analysis (B, C, F and G).

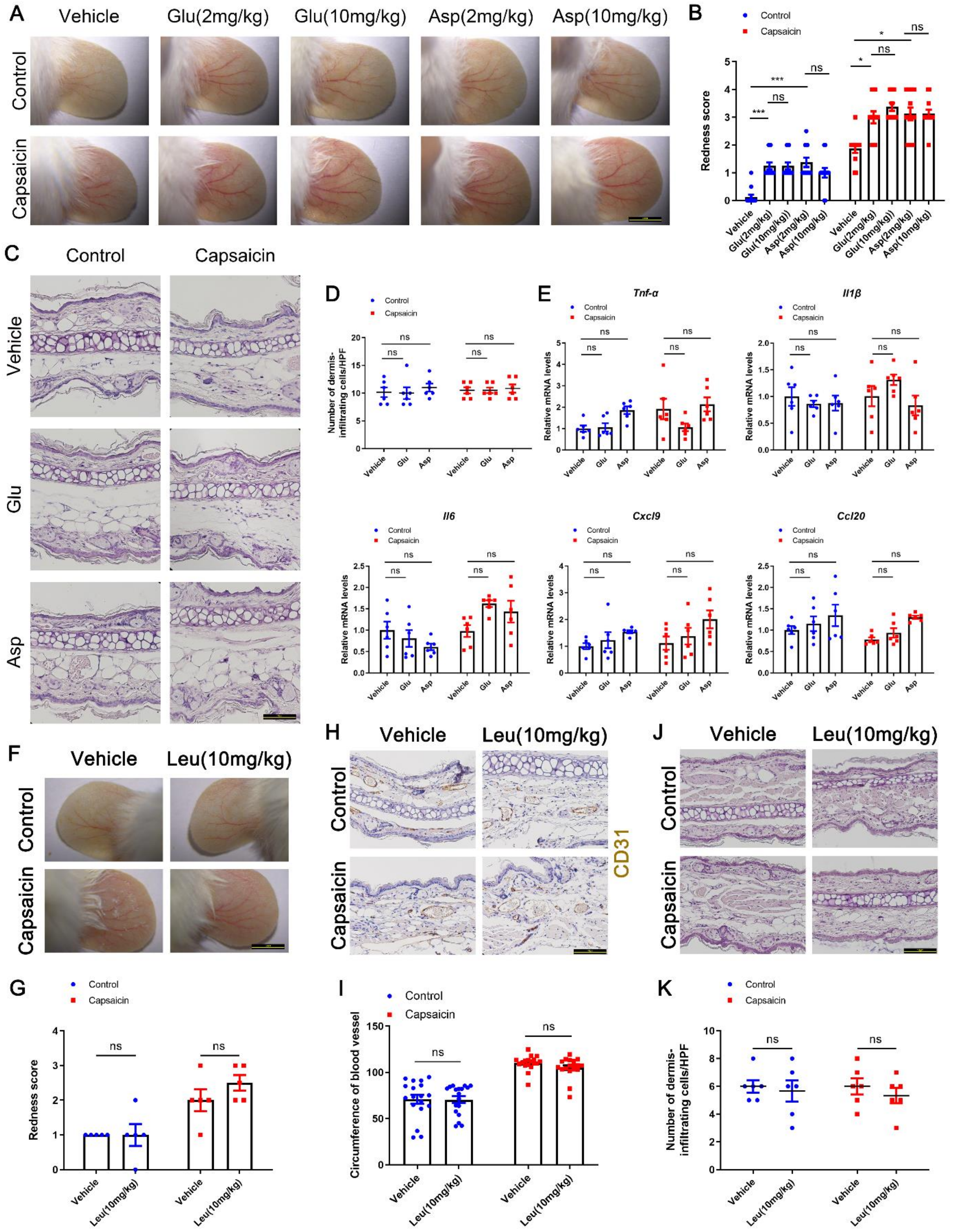

Supplemental Figure 2. Glutamic acid and aspartic acid promote rosacea-like erythema and vasodilation in mice.

(**A**) The ear of mice treated with different dose of glutamic acid and aspartic acid were smeared

with capsaicin or control vehicle (n = 6 for each group). Images were taken 30 min after capsaicin administration. Scale bar, 2mm (**B**) The severity of the rosacea-like phenotype was assessed on account of the redness score. (n=12 for each group). (**C**) HE staining of ear sections from different groups. (n = 6 for each group) (**D**) Dermal infiltrating cells were quantified (n = 6 for each group). (**E**) The mRNA expression levels of *Tnf-α, Il1β, Il6, Cxcl9, Ccl20* in ears. (**F**) The ear of mice treated with leucine were smeared with capsaicin or control vehicle (n = 5 for each group). Images were taken 30 min after capsaicin administration. Scale bar, 2mm. (**G**) The severity of the rosacea-like phenotype was assessed on account of the redness score (n=5 for each group). (**H**) IHC of CD31 on ear sections from mice. Scale bar, 100μm. (**I**) The circumference of blood vessels in each group was calculated (n=6 mice for each group). (**J**) HE staining of ear sections from different groups (n = 6 for each group). (**K**) Dermal infiltrating cells were quantified (n = 6 for each group). (Data represent the mean ± SEM. *P < 0.05, **P < 0.01, ***P < 0.001. ns, not significant. One-way ANOVA with Bonferroni's *post hoc* test was used (B, D, E, G, I and K).

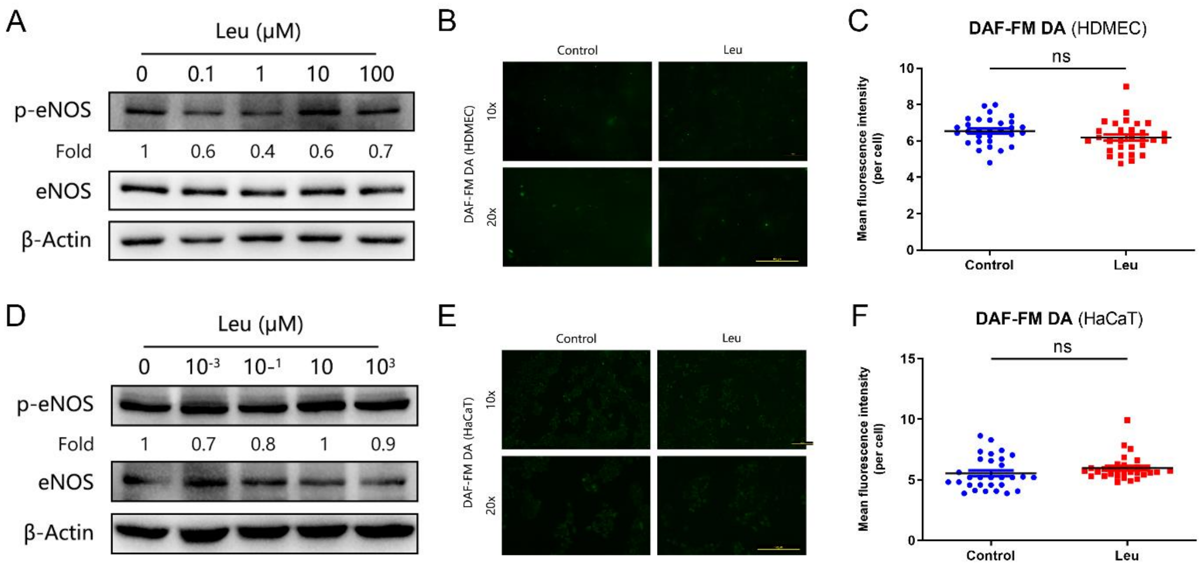

Supplemental Figure 3. Leucine fails to stimulate the production of nitric oxide from endothelial cells and keratinocytes. (**A**) Immunoblot analysis of the p-eNOS and total eNOS in cell lysates from HDMEC after leucine treatment. β-Actin is the loading control. p-eNOS protein levels were analyzed relative to total eNOS. (**B**) DAF-FM DA staining in HDMEC after leucine treatment. Scale bar, 100μm. (**C**) Quantification of intensity of DAF-FM DA fluorescence in HDMEC

under the designated treatments (n=30 cells). The quantification results are representative of at least 3 independent experiments. (**D**) Immunoblot analysis of the p-eNOS and total eNOS in cell lysates from HaCaT keratinocytes after leucine treatment. β-Actin is the loading control. p-eNOS protein levels were analyzed relative to total eNOS. (**E**) DAF-FM DA staining in HaCaT keratinocytes after leucine treatment. Scale bar, 100μm. (**F**) Quantification of intensity of DAF-FM DA fluorescence in HaCaT keratinocytes under the designated treatments (n=30 cells). The quantification results are representative of at least 3 independent experiments. All results are representative of at least three independent experiments. Data represent the mean ± SEM. **P < 0.01, ***P < 0.001. Two-tailed unpaired Student's t-test was used (C and F).

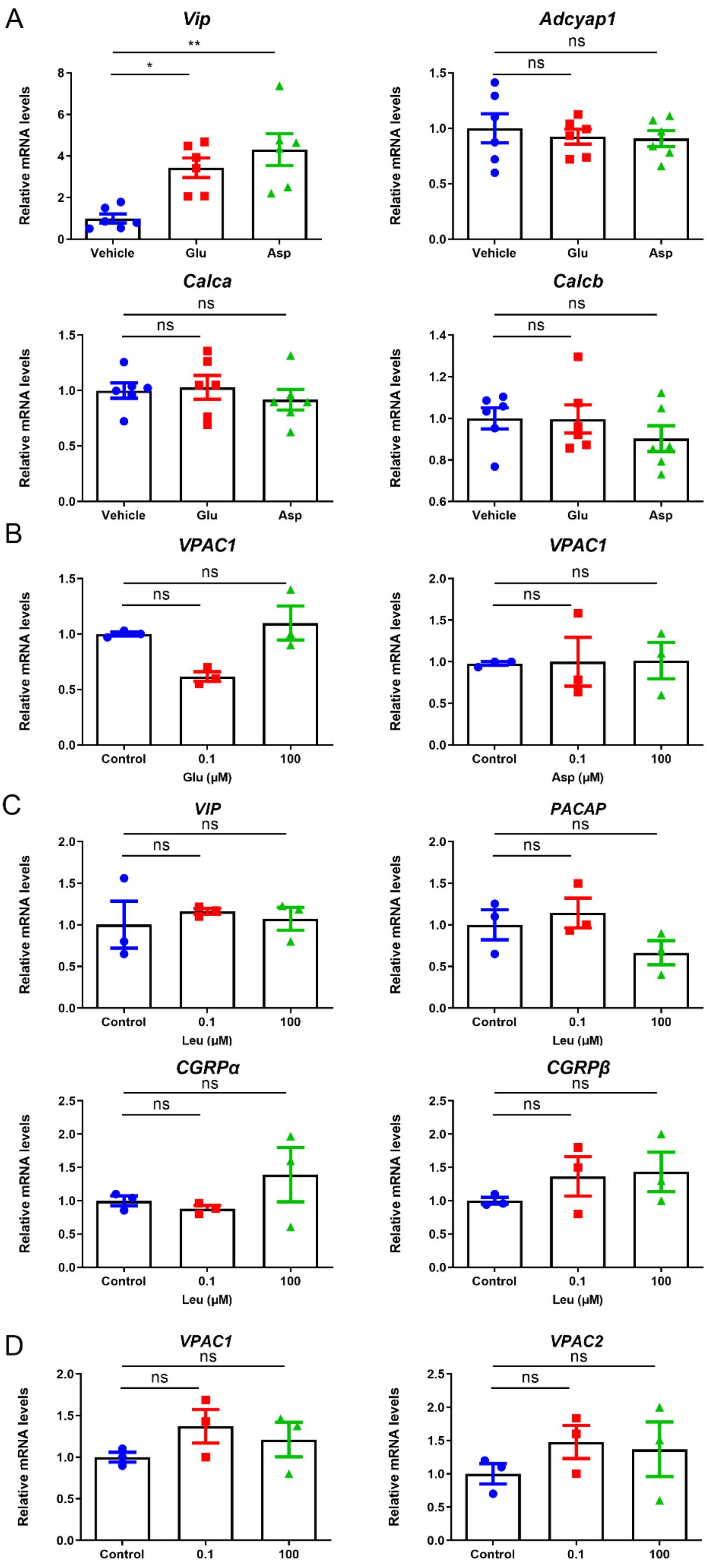

A
Vip
**
*
Relative mRNA levels
Vehicle
Glu
Asp
Adcyap1
ns
ns
Calca
ns
ns
Calcb
ns
ns
B
VPAC1
ns
ns
Control
0.1
100
Glu (μM)
VPAC1
ns
ns
Asp (μM)
C
VIP
ns
ns
Leu (μM)
PACAP
ns
ns
Leu (μM)
CGRPα
ns
ns
Leu (μM)
CGRPβ
ns
ns
Leu (μM)
D
VPAC1
ns
ns
VPAC2
ns
ns

Supplemental Figure 4. Glutamic acid and aspartic acid induce vasodilation-related neuropeptides from peripheral neuron.

(**A**) The mRNA expression levels of *Vip, Adcyap1, Calca* and *Calcb* in DRG neurons from glutamic acid or aspartic acid gavage mice and control group. (**B**) The mRNA expression levels of *VPAC1* in HDMEC after glutamic acid or aspartic acid treatment. (**C**) The mRNA expression levels of *VIP, PACAP, CGRPα* and *CGRPβ* in HaCaT keratinocytes after leucine treatment (n=3 for each group). (**D**) The mRNA expression levels of *VPAC1* and *VPAC2* in HDMEC after leucine treatment (n=3 for each group). Data represent the mean ± SEM. $^*P < 0.05$, $^{**}P < 0.01$, ns, not significant. One-way ANOVA with Bonferroni's *post hoc* test was used (A-D).

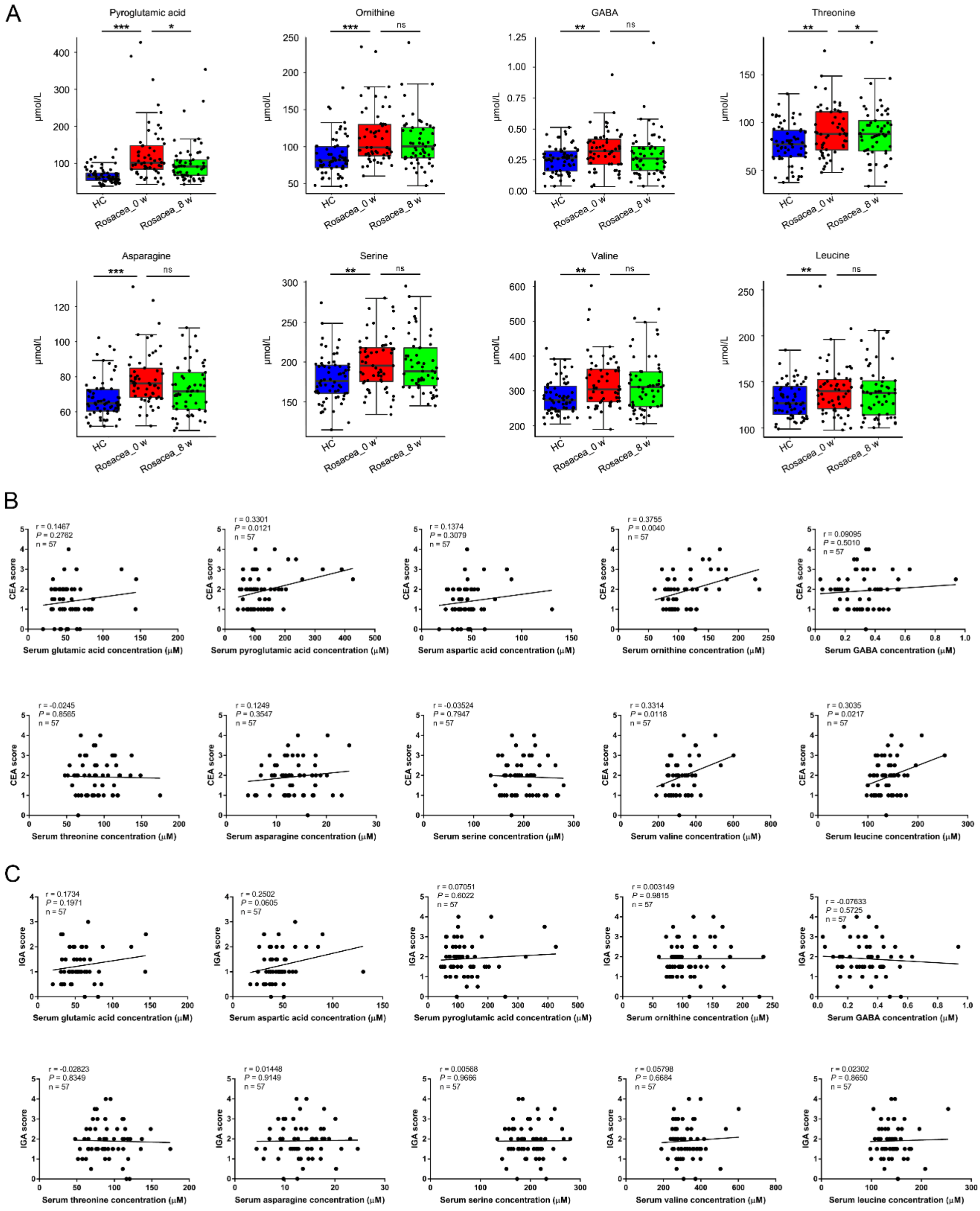

Supplemental Figure 5. Expression changes of amino acids in serum after doxycycline treatment in patients with rosacea.

(**A**) Boxplot of serum levels of top 10 differential amino acids in different groups except glutamic acid and aspartic acid. (**B**) Correlation of serum amino acid levels in rosacea patients after

doxycycline treatment with CEA scores. (**C**) Correlation of serum amino acid levels in rosacea patients after doxycycline treatment with IGA scores. Data represent the mean ± SEM. *$P < 0.05$, **$P < 0.01$, ***$P < 0.001$. One-way ANOVA with Bonferroni's *post hoc* test was used (A). Spearman's correlation test was used for the correlation analysis (B and C).

Supplementary Table 1. Classification and expression changes of differential metabolites between rosacea patients and healthy controls.

Class	Metabolite	HMDB	*P*-value	log2FC
Amino Acids (30.2%)	Arginine	HMDB0000517	0.03331	-0.1225
	Asparagine	HMDB0000168	1.8E-06	0.2305
	Aspartic acid	HMDB0000191	6.7E-11	0.6028
	GABA	HMDB0000112	0.00759	0.2756
	Glutamic acid	HMDB0000148	7.9E-13	0.6834
	Histidine	HMDB0000177	0.0013	0.0871
	Leucine	HMDB0000687	0.02919	0.1417
	Lysine	HMDB0000182	0.04359	0.0646
	Ornithine	HMDB0000214	5.5E-06	0.2772
	Pyroglutamic acid	HMDB0000267	7E-11	0.6954
	Serine	HMDB0000187	0.00059	0.1539
	Threonine	HMDB0000167	0.00228	0.2308
	Valine	HMDB0000883	0.001	0.1521
Benzenoid (2.3%)	Phenylpyruvic acid	HMDB0000205	8E-05	-0.6661
Carbohydrates (16.3%)	Erythronic acid	HMDB0000613	3.2E-15	0.9981
	Glucose	HMDB0000122	0.01002	-0.0975
	Glyceric acid	HMDB0000139	3.2E-14	0.6847
	N-Acetylneuraminic acid	HMDB0000230	1.9E-06	0.3594
	Ribulose	HMDB0000621	1.9E-05	0.3349
	Threonic acid	HMDB0000943	9E-12	1.2350
	Xylulose	HMDB0001644	5.3E-05	0.3887
Carnitines (2.3%)	Oleylcarnitine	HMDB0005065	0.03038	0.3094
Fatty Acids	10Z-Heptadecenoic acid	HMDB0060038	0.00058	0.5959
	Adrenic acid	HMDB0002226	9.9E-05	0.4812
	Arachidonic acid	HMDB0001043	1.8E-09	0.4926
	Citraconic acid	HMDB0000634	0.0011	-0.1043
	DHA	HMDB0002183	0.00112	0.2879
	Dihomo-gamma-linolenic	HMDB0002925	9.4E-06	0.5602

(27.9%)	acid			
	DPAn-6	HMDB0001976	0.00509	0.6561
	gamma-Linolenic acid	HMDB0003073	0.00167	0.2942
	Linoleic acid	HMDB0000673	0.00012	0.3606
	Myristic acid	HMDB0000806	0.00088	0.4523
	Oleic acid	HMDB0000207	0.00222	0.2203
	Palmitoleic acid	HMDB0003229	0.00041	0.4569
Organic Acids (18.6%)	2-Hydroxyglutaric acid	HMDB0000606	1.9E-05	0.6511
	Aconitic acid	HMDB0000072	0.00048	0.2316
	Fumaric acid	HMDB0000134	0.0024	0.5928
	Lactic acid	HMDB0000190	3.2E-10	0.4535
	Maleic acid	HMDB0000176	7.7E-06	0.4856
	Malic acid	HMDB0000156	8E-11	0.7251
	Oxoglutaric acid	HMDB0000208	0.00067	0.2250
	Succinic acid	HMDB0000254	0.00462	0.2224
Phenols (2.3%)	Homovanillic acid	HMDB0000118	0.00274	0.2393

Supplementary Table 2. Expression changes of differential amino acids between rosacea patients and healthy controls from the validation cohort.

Class	Metabolite	HMDB	*P*-value	log2FC
Amino Acids	Arginine	HMDB0000517	0.0438	-0.2691
	Aspartic acid	HMDB0000191	3.55E-05	0.4411
	Citrulline	HMDB0000904	0.01115	-0.2542
	Glutamic acid	HMDB0000148	0.00015	0.4879
	Phenylalanine	HMDB0000159	0.00035	0.1524
	Serine	HMDB0000187	0.00749	0.1458
	Tryptophan	HMDB0000929	0.03176	-0.1317

Supplementary Table 3. Classification and expression changes of differential metabolites between 8 weeks and 0 weeks after doxycycline treatment in rosacea patients.

Class	Metabolite	HMDB	*P*-value	log2FC
Amino Acids (21.1%)	Asparagine	HMDB0000168	0.04668	-0.0889
	Aspartic acid	HMDB0000191	0.00126	-0.2027
	Glutamic acid	HMDB0000148	0.00512	-0.232
	Pyroglutamic acid	HMDB0000267	0.01306	-0.1536
Benzoic Acids (5.3%)	m-Aminobenzoic acid	HMDB0001891	0.04544	0.0946
Bile Acids	CA	HMDB0000619	0.02557	-0.8291

(10.5%)	UDCA	HMDB0000946	0.03912	-0.3128
Carbohydrates (31.6%)	Erythronic acid	HMDB0000613	0.0419	-0.1993
	Fructose	HMDB0000660	0.01094	-0.2864
	Glyceric acid	HMDB0000139	0.01306	-0.238
	N-Acetylneuraminic acid	HMDB0000230	0.01245	-0.215
	Tartaric acid	HMDB0000956	0.00105	0.2597
	Threonic acid	HMDB0000943	0.02997	-0.1824
Organic Acids (26.3%)	Lactic acid	HMDB0000190	0.01059	-0.2131
	Maleic acid	HMDB0000176	0.0062	-0.3086
	Malic acid	HMDB0000156	0.02519	-0.3026
	Oxoglutaric acid	HMDB0000208	0.031278	-0.2385
	Pyruvic acid	HMDB0000243	0.03218	-0.2423
SCFAs (5.3%)	Isocaproic acid	HMDB0000689	0.04464	-0.2025

Supplementary Table 4. Differentially-expressed, doxycycline-target genes associated with glutamic acid and aspartic acid by multiomics analysis.

Genes	Target	*P*-value	log2FC
ADORA2A	Glutamic acid	0.01317	1.171
AKT1	Glutamic acid	0.00135	2.3883
ANPEP	Glutamic acid	1.08E-07	-6.8843
APH1A	Aspartic acid	5.91E-05	-3.2804
BAX	Glutamic acid	1.67E-06	-4.5183
BCL2	Aspartic acid<br>Glutamic acid	5.53E-06	3.8918
BST1	Glutamic acid	0.00368	4.3019
CBS	Glutamic acid	0.0011	2.2627
CDK5	Glutamic acid	3.39E-06	-5.8171
CHRNA4	Glutamic acid	0.00057	3.1444
COMT	Glutamic acid	0.00053	-2.2979
CYCS	Glutamic acid	5.38E-06	-3.4732
DHFR	Glutamic acid	0.00101	1.9473
DNPEP	Glutamic acid	2.01E-06	-4.9377
FGFR1	Glutamic acid	4.55E-06	6.4554
FOS	Glutamic acid	0.01467	-2.6362
GRIA2	Aspartic acid<br>Glutamic acid	2.51E-05	4.8757
GRIN1	Aspartic acid<br>Glutamic acid	0.00012	3.7986
GRM5	Glutamic acid	0.00011	3.297

GSTO1	Glutamic acid	3.2E-06	-5.7778
GSTP1	Glutamic acid	0.00603	-1.1703
IL6	Glutamic acid	4.8E-07	7.475
KMO	Glutamic acid	0.00116	-1.9304
LDHB	Aspartic acid<br>Glutamic acid	0.0056	-2.0407
LYZ	Aspartic acid	4.6E-05	-3.4105
MMP2	Glutamic acid	0.00011	-8.1293
NMNAT3	Glutamic acid	5.99E-06	-4.7931
NOS2	Glutamic acid	0.00854	5.1937
NR3C1	Glutamic acid	9.77E-06	-7.3827
OTC	Aspartic acid<br>Glutamic acid	9.13E-05	-5.8441
PLA2G1B	Glutamic acid	4.2E-05	5.5922
PSEN1	Aspartic acid	0.00729	-1.5423
SIGMAR1	Glutamic acid	2.65E-08	-9.0496
SLC6A1	Glutamic acid	4.46E-07	7.2995
SOD1	Glutamic acid	2.46E-07	-5.9784
SYN1	Glutamic acid	0.00137	-2.4099
TACR1	Glutamic acid	3.38E-06	-6.1159
TGFB2	Glutamic acid	9.21E-07	8.0598
TGM2	Glutamic acid	8.09E-08	-7.4568
TP53	Glutamic acid	5.02E-07	-6.2877
TRPV1	Glutamic acid	1.45E-06	7.0917

Supplementary Table 5

qPCR primers	
Human-*GAPDH*-F	TGTTGCCATCAATGACCCCTT
Human-*GAPDH*-R	CTCCACGACGTACTCAGCG
Human-*VIP*-F	GACACCAGAAATAAGGCCCAG
Human-*VIP*-R	GTCACCCAACCTGAGAGCAG
Human-*PACAP*-F	CAGACCCTGAGAACCTACGC
Human-*PACAP*-R	CTGGCTGGTTTGTCTGTTGC
Human-*CGRPα*-F	AAGCGGTGCGGTAATCTGAG
Human-*CGRPα*-R	GGGGAACGTGTGAAACTTGTTG
Human-*CGRPβ*-F	ACCCGGCCACACTCAGTAA
Human-*CGRPβ*-R	GGGCACGAAGTTGCTCTTCA
Human-*VPAC1*-F	TCATCCGAATCCTGCTTCAGA

Human-*VPAC1*-R	AGGCGAACATGATGTAGTGTACT
Human-*VPAC2*-F	CAGTGGCGTCTGGGACAAC
Human-*VPAC2*-R	CCGTCACTCGTACAGTTTTTGC
Mouse-*Gapdh*-F	AGGTCGGTGTGAACGGATTTG
Mouse-*Gapdh*-R	TGTAGACCATGTAGTTGAGGTCA
Mouse-*Vip*-F	AGTGTGCTGTTCTCTCAGTCG
Mouse-*Vip*-R	GCCATTTTCTGCTAAGGGATTCT
Mouse-*Adcyap1*-F	ACCATGTGTAGCGGAGCAAG
Mouse-*Adcyap1*-R	CTGGTCGTAAGCCTCGTCT
Mouse-*Calca*-F	GAGGGCTCTAGCTTGGACAG
Mouse-*Calca*-R	AAGGTGTGAAACTTGTTGAGGT
Mouse-*Calcb*-F	CTCTCAGCACGATATGGGTCC
Mouse-*Calcb*-R	GCAAGAGATGTTTTTCCTGGTCG
Mouse-*Tnf-α*-F	CCCTCACACTCAGATCATCTTCT
Mouse-*Tnf-α*-R	GCTACGACGTGGGCTACAG
Mouse-*Il1β*-F	GCAACTGTTCCTGAACTCAACT
Mouse-*Il1β*-R	ATCTTTTGGGGTCCGTCAACT
Mouse-*Il6*-F	TAGTCCTTCCTACCCCAATTTCC
Mouse-*Il6*-R	TTGGTCCTTAGCCACTCCTTC
Mouse-*Cxcl9*-F	GGAGTTCGAGGAACCCTAGTG
Mouse-*Cxcl9*-R	GGGATTTGTAGTGGATCGTGC
Mouse-*Ccl20*-F	GCCTCTCGTACATACAGACGC
Mouse-*Ccl20*-R	CCAGTTCTGCTTTGGATCAGC

Full unedited blot for Figure 4D

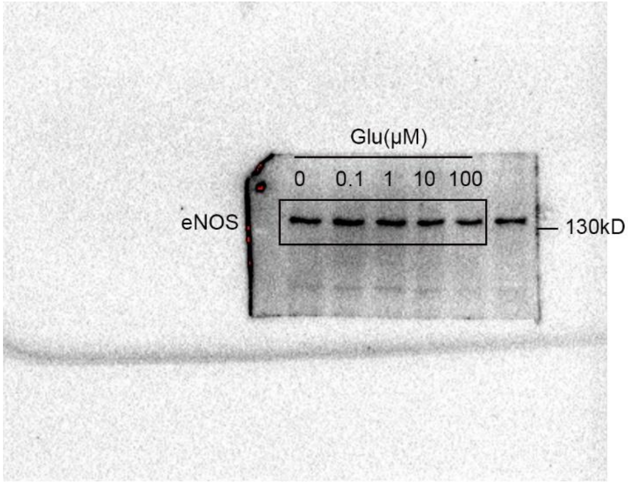

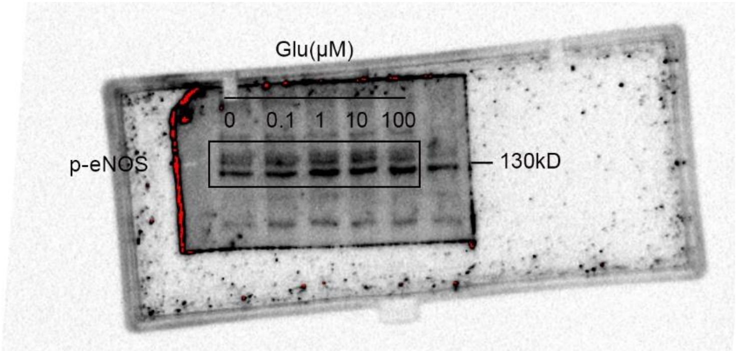

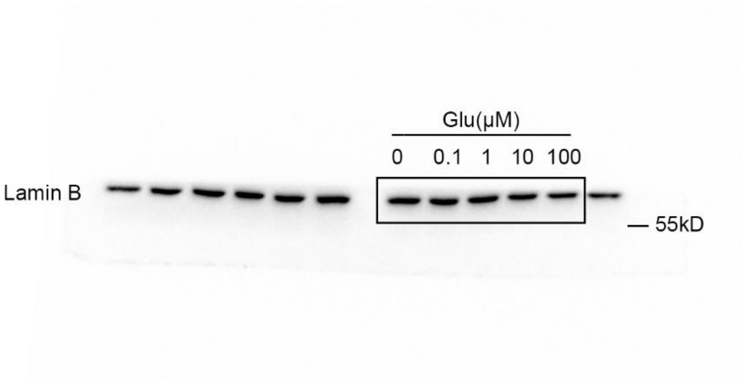

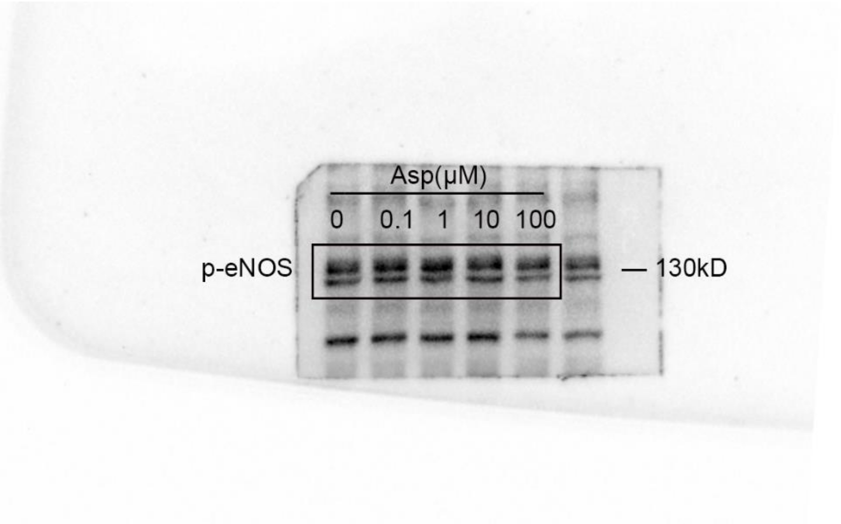
Asp(μM)
0 0.1 1 10 100
p-eNOS
— 130kD

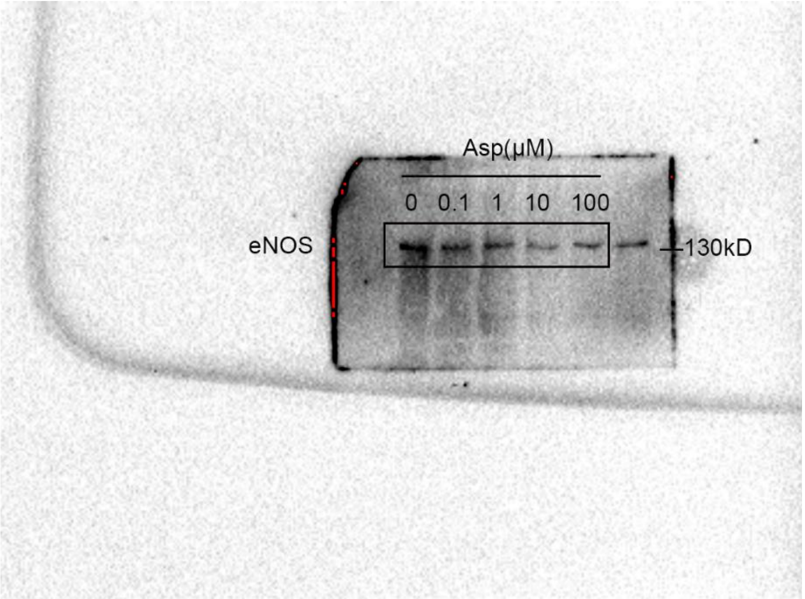
Asp(μM)
0 0.1 1 10 100
eNOS
—130kD

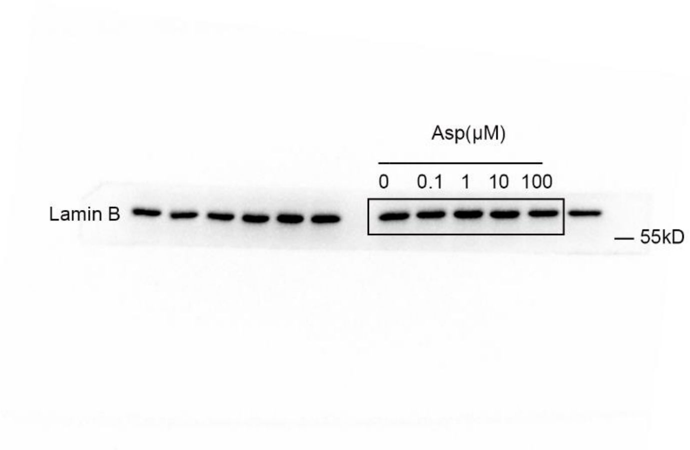
Asp(μM)
0 0.1 1 10 100
Lamin B
— 55kD

Full unedited blot for Figure 4G

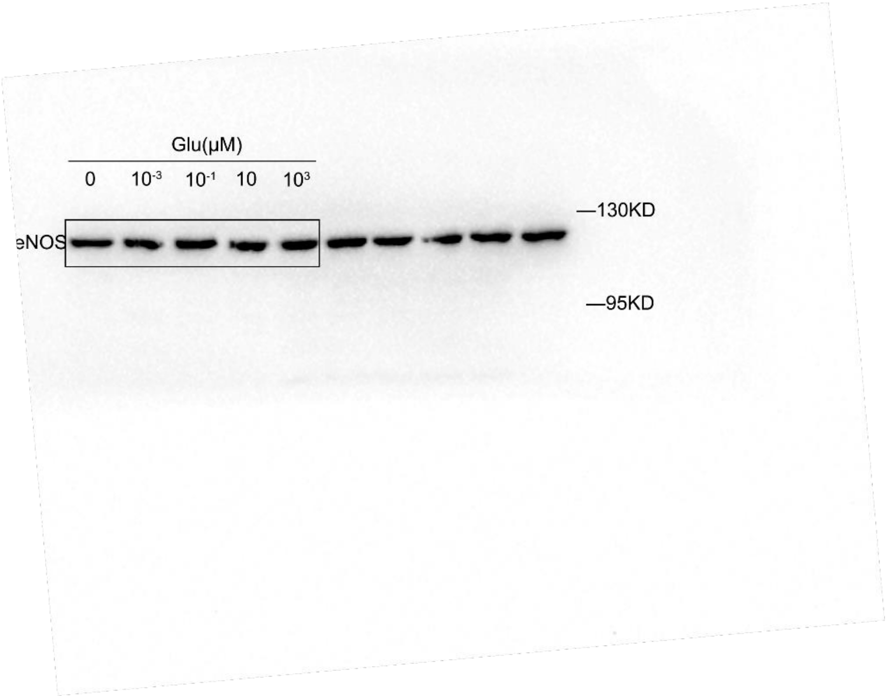

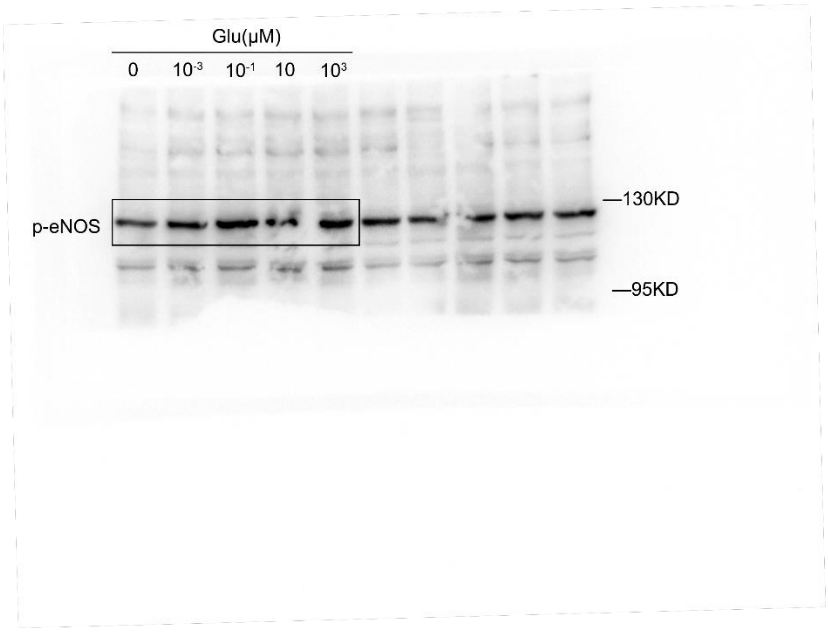

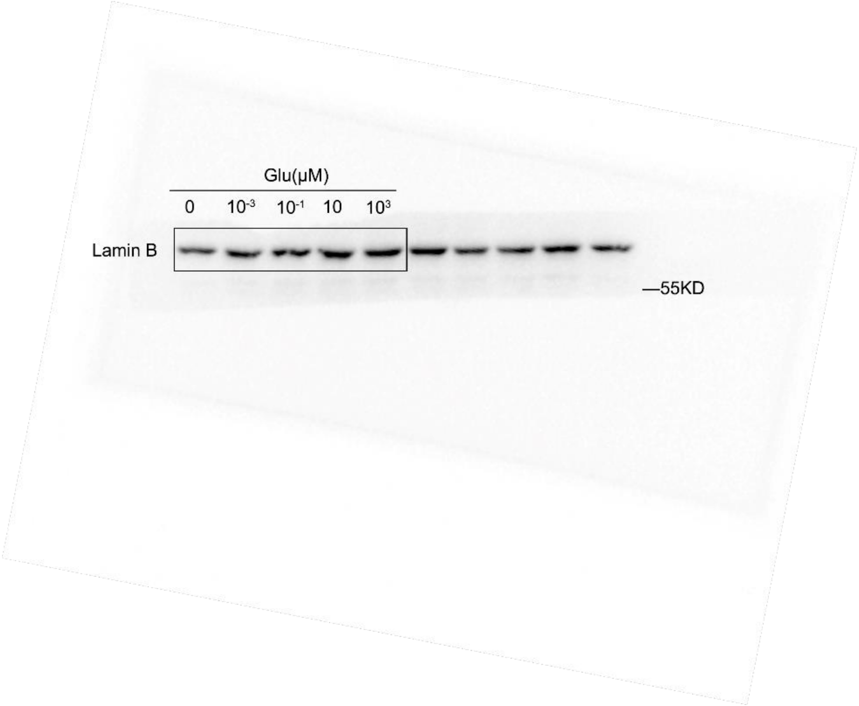
Glu(μM)
0
10-3
10-1
10
103
Lamin B
—55KD

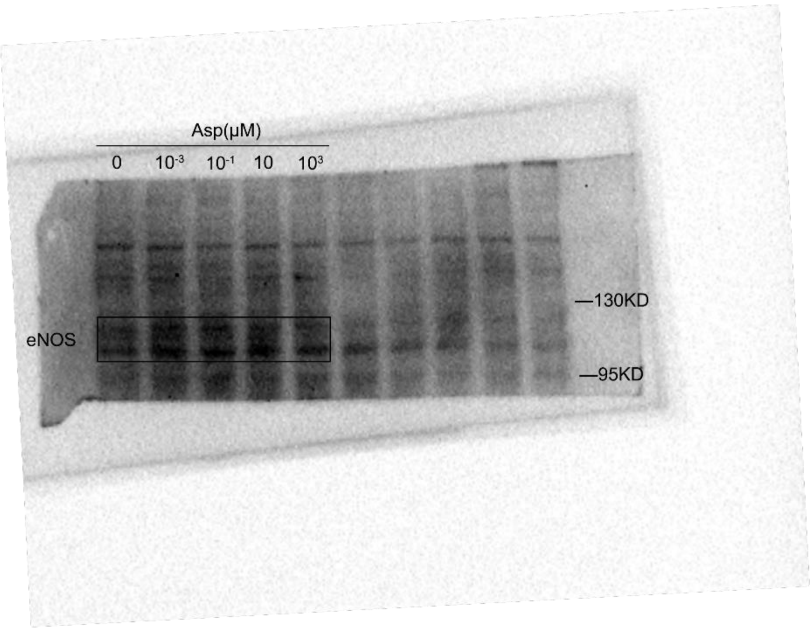
Asp(μM)
0
10-3
10-1
10
103
eNOS
—130KD
—95KD

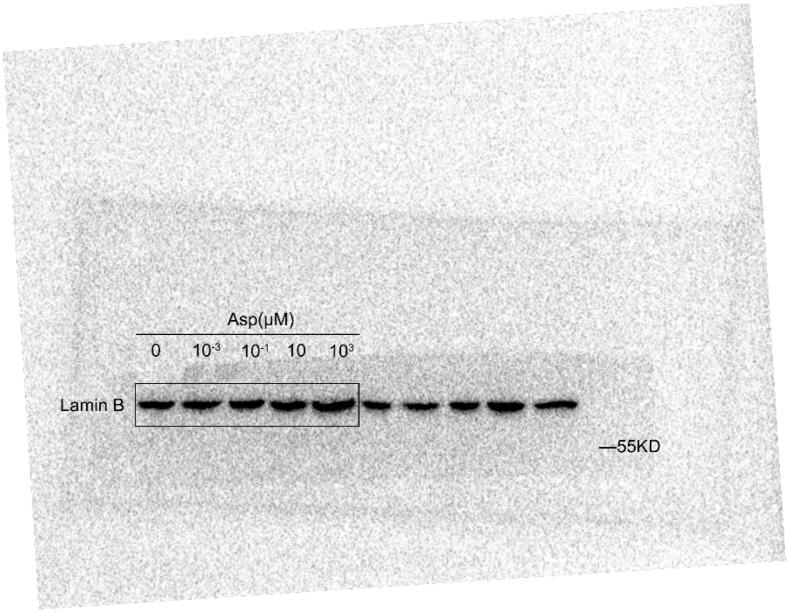

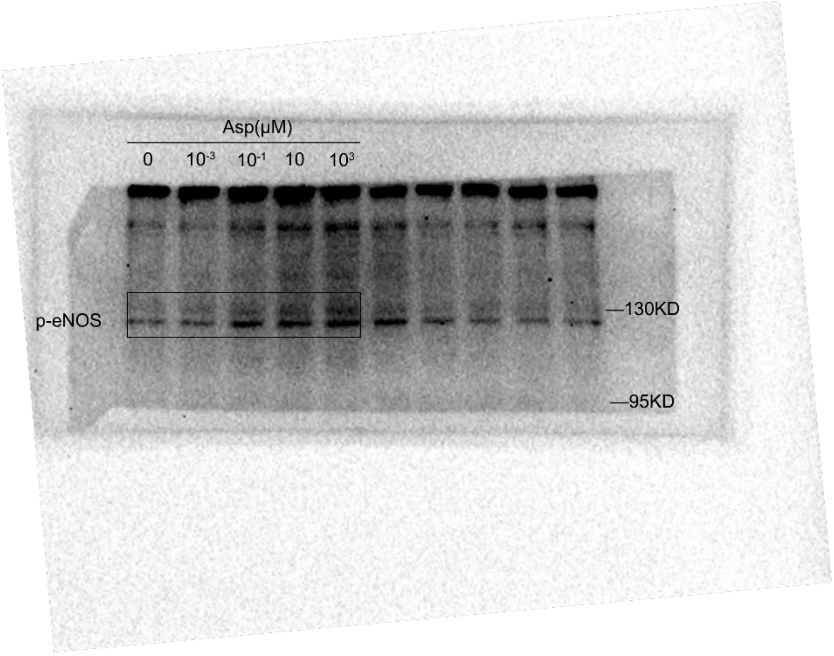

Full unedited blot for Figure 5A

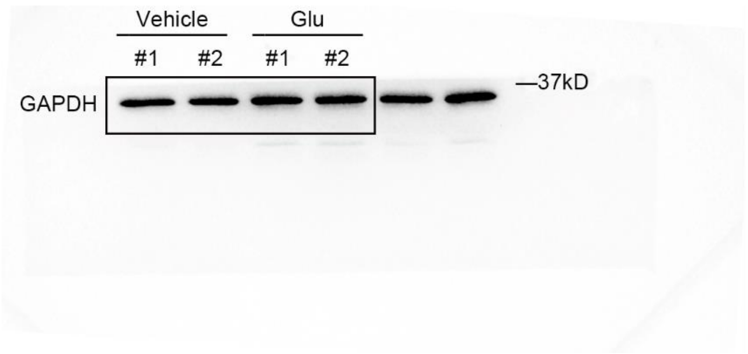

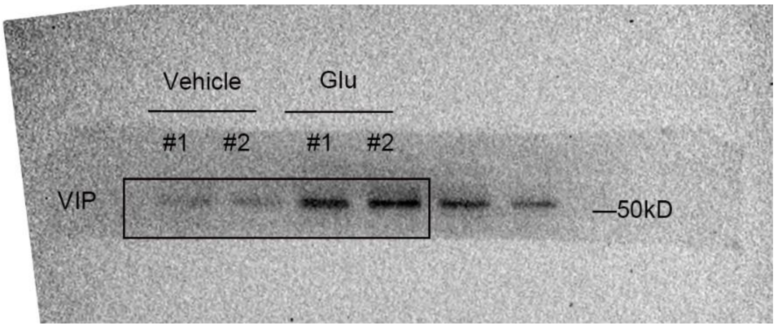

Full unedited blot for Figure 5B

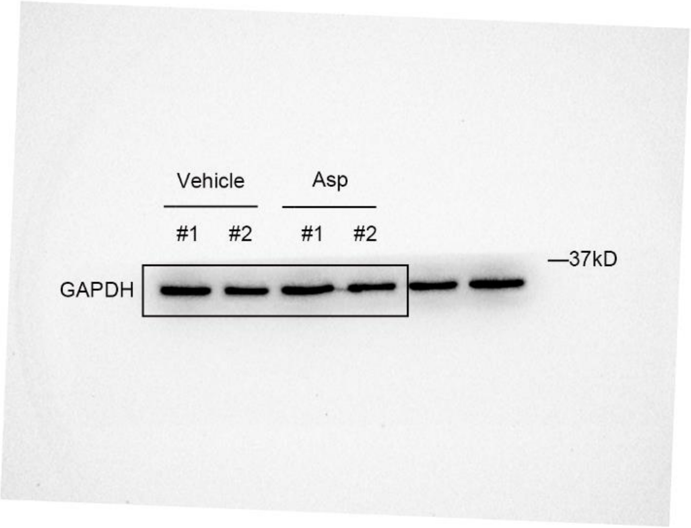

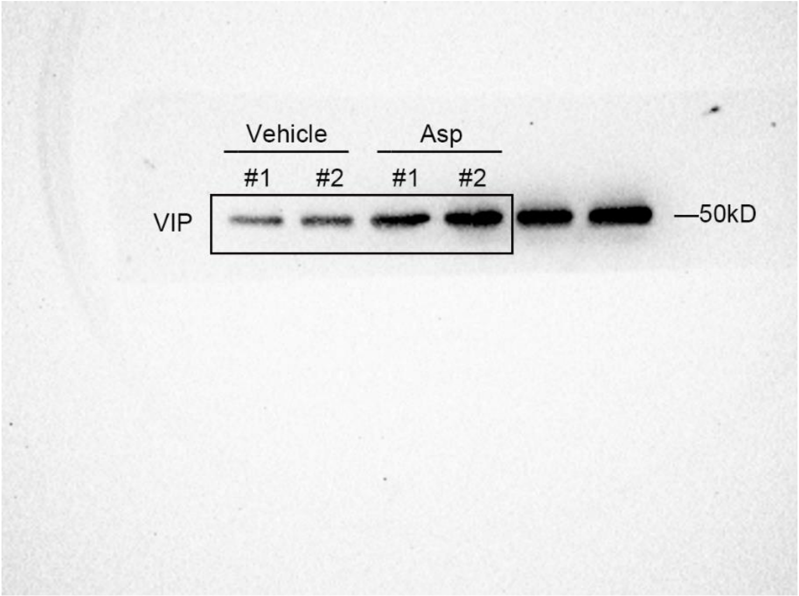

Full unedited blot for Figure 5F

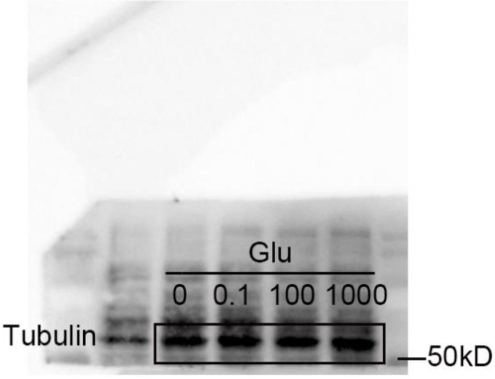

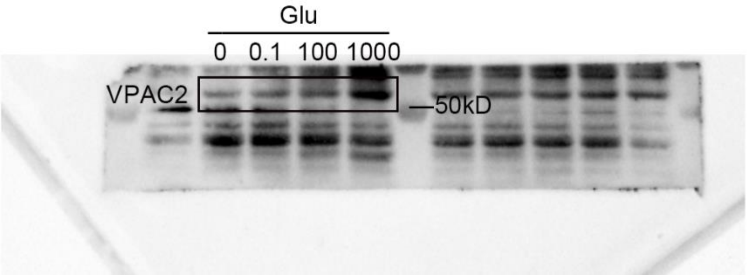

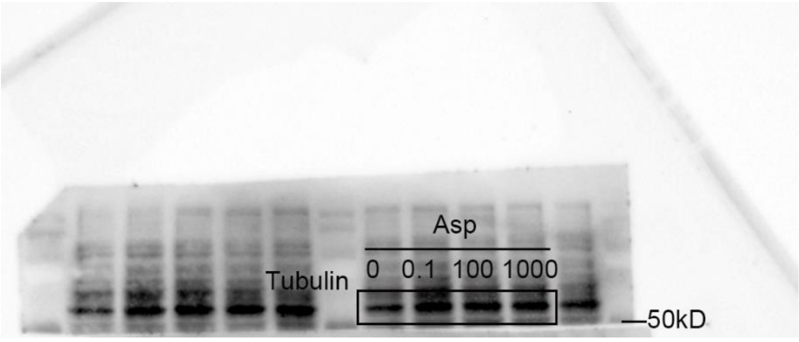

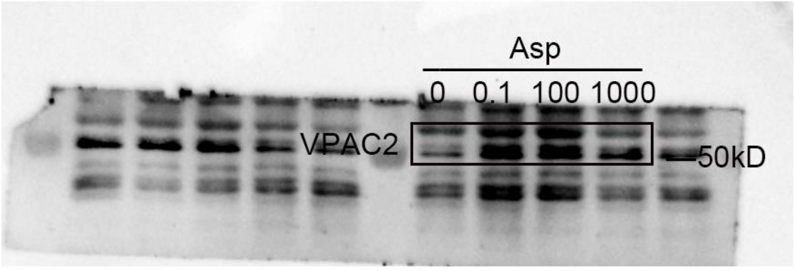

Full unedited blot for Supplementary Figure 3A

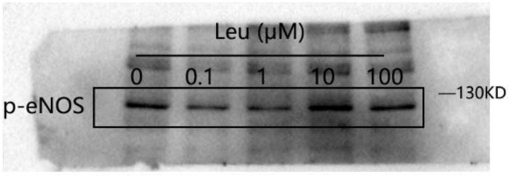

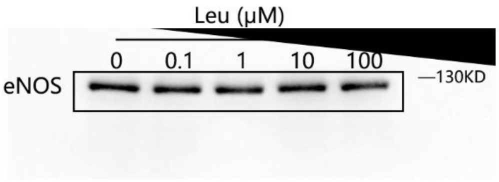

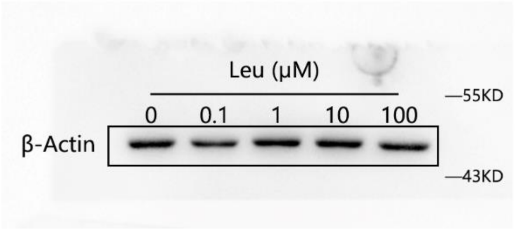

Full unedited blot for Supplementary Figure 3D

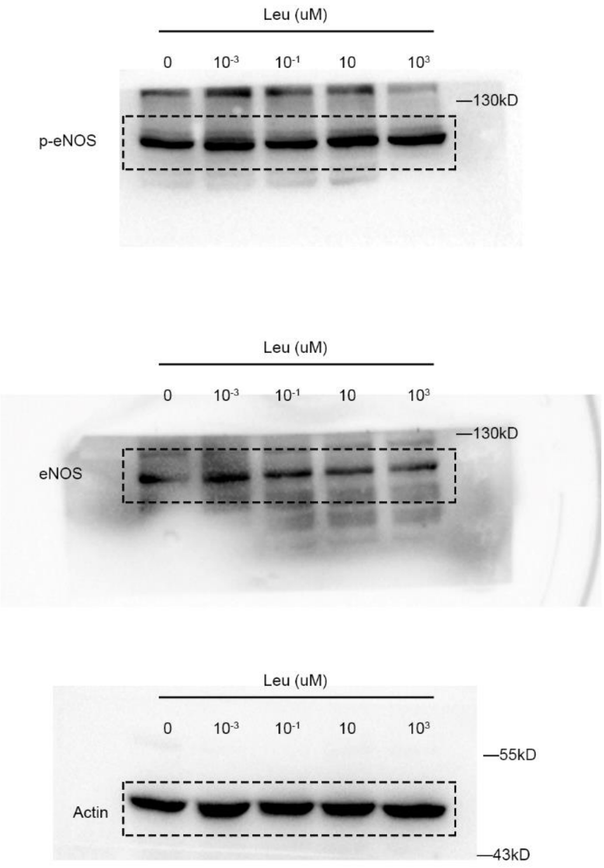

Leu (uM)
0
10-3
10-1
10
103
—130kD
p-eNOS
Leu (uM)
0
10-3
10-1
10
103
—130kD
eNOS
Leu (uM)
0
10-3
10-1
10
103
—55kD
Actin
—43kD
